# The most promising microneedle device: present and future of hyaluronic acid microneedle patch

**DOI:** 10.1080/10717544.2022.2125600

**Published:** 2022-09-23

**Authors:** Huizhi Kang, Zhuo Zuo, Ru Lin, Muzi Yao, Yang Han, Jing Han

**Affiliations:** a Department of Pharmaceutical Engineering, Shenyang Pharmaceutical University, Shenyang, China; b School of Chinese Materia Medica, Shenyang Pharmaceutical University, Shenyang, China; c Faculty of Functional Food and Wine, Shenyang Pharmaceutical University, Shenyang, China

**Keywords:** Microneedle patch, hyaluronic acid, TDDS, drug delivery, extraction

## Abstract

Microneedle patch (MNP) is an alternative to the oral route and subcutaneous injection with unique advantages such as painless administration, good compliance, and fewer side effects. Herein, we report MNP as a prominent strategy for drug delivery to treat local or systemic disease. Hyaluronic acid (HA) has advantageous properties, such as human autologous source, strong water absorption, biocompatibility, and viscoelasticity. Therefore, the Hyaluronic acid microneedle patch (HA MNP) occupies a large part of the MNP market. HA MNP is beneficial for wound healing, targeted therapy of certain specific diseases, extraction of interstitial skin fluid (ISF), and preservation of drugs. In this review, we summarize the benefits of HA and cross-linked HA (x-HA) as an MNP matrix. Then, we introduce the types of HA MNP, delivered substances, and drug distribution. Finally, we focus on the biomedical application of HA MNP as an excellent drug carrier in some specific diseases and the extraction and analysis of biomarkers. We also discuss the future development prospect of HA MNP in transdermal drug delivery systems (TDDS).

## 1. Introduction

Since the FDA approved the first scopolamine transdermal patch (Transdermal-Scop^®^) in 1979 (Samad et al., 2009), there has been a surge in the use of transdermal drug delivery systems (TDDS). TDDS has advantages over oral administration and subcutaneous injection in terms of avoiding first-pass effects, reducing injection pain, controlling and stabling plasma concentration, and improving patient compliance (Gowda et al., 2022; Sabbagh and Kim, 2022). However, the skin stratum corneum (SC), which is about 10–20 μm thick (Al-Zahrani et al., 2012; Teo et al., 2006), has special components and a dense structure, hence is the largest barrier to transdermal drug permeation (Brown and Langer, 1988). In the past decades, with the continuous improvement and refinement of science and technology, various chemical and physical methods (Table 1) have been explored to improve skin permeability or to provide a driving force for drugs to achieve the goal of enhancing drug transportation in the skin (Ahmadi-Ashtiani et al., 2020; Dragicevic and Maibach, 2018).

**Table 1.** List of three current methods of promoting permeation for TDDS.

Method	Concrete content	Reference
Physical penetration	Ion introduction, electroporation, ultrasonic introduction	Lavon and Kost, 2004
Chemical penetration	Fatty acids, azones, alcohols, amines, terpenes	Yang et al., 2011
Pharmacy	Liposomes, transfersomes, ethosomes, microemulsions	Dragicevic and Maibach, 2018; Zhang et al., 2020a

However, these approaches are considerably limited in terms of practical application and economics. MNP can overcome these challenges by enhancing drug penetration and bioavailability in the skin. The uniqueness of MNP allows them to combine the advantages of both subcutaneous injection and topical transdermal patches. MNP is long enough to penetrate the dermis, yet short and narrow enough to avoid irritating dermal nerves or piercing dermal blood vessels. Needle bodies are typically several hundred microns in diameter, the length typically ranges from 100 to 1000 μm, and tip sizes range from 1 to 10 μm (Prausnitz, 2017). The length and number of microneedles (MNs) depend on the intended purpose. MNs differ from conventional subcutaneous injections in two ways: 1) The pinholes are tiny, therefore relatively painless; 2) drug transdermal transport relies on diffusion-driven by chemical potential differences, rather than convection driven by pressure differences.

The concept of using MNs for drug delivery dates back to 1976, and the first concept of MNP for TDDS was in a US patent describing the formation of micropores in the skin to improve drug penetration without causing pain. Following this, MNs technology has been extensively investigated using a variety of materials and designs. For example, hierarchical MNP with multifunctional adhesion and antimicrobial capabilities inspired by the adhesion mechanisms of mussel and octopus tentacles were developed (Zhang et al., 2020c). Then, for example, a novel serration-like clamping MNP based on ferromagnetic fluid configuration molding was proposed, which was inspired by the serrated microstructure of mantis forelimbs (Zhang et al., 2019). Now MNs can be used not only for small molecule drugs but also for transdermal transfer of biomolecules such as hormones (Naito et al., 2018; Nguyen et al., 2022), proteins (Katsumi et al., 2014), vaccines (Zhou et al., 2022), and genetic material (Wang et al., 2020). With the development of new smart microneedle systems, the field of MNs for improving drug and cosmeceutical penetration is growing exponentially. The market for MNP is forecasted to grow at a compound annual growth rate (CAGR) of 66% from 2020 to 2035, with drug sales via MNP reaching $1,294 million in 2035. There are few medicinal MNs products currently on the market. Valeritas has introduced a V-Go (essentially hollow MNs), a disposable insulin injection device designed for adults with type II diabetes. There are also coin-sized MNs prepared by Minnesota Mining and Manufacturing Corporation (3M) using Class IV medical polymers, which are currently used for the vaccine (for children) and insulin (for diabetics) delivery (https://www.qyresearch.com/market-reports-list).

In terms of MNs materials and development, MNs can be classified into four generations. The first generation was solid MNs (solid MNs made from silicon and silicon oxide) (Prausnitz, 2004; Qiu et al., 2008; Teo et al., 2006), and the second generation was metallic MNs, and the third generation was dissolving MNs (prepared from polymeric polymers). The fourth generation is the recent development of hydrogel MNs (hydrogel MNs prepared from substrates that swell and do not dissolve in water) (Donnelly et al., 2012), such as those prepared from methacrylated hyaluronic acid (MeHA). According to the MNs mechanism, MNs can be specifically classified as solid MNs ‘poke and patch’, coated MNs ‘coat and poke’, hollow MNs ‘poke and flow’, and hydrogel MNs ‘poke and release’. From simple first generation MNs to fourth generation MNs, MNs have become painless, safe, effective, self-administered, avoided enzymatic degradation and reduced systemic toxicity, dose saving, etc. As a proof concept, the first and second generation inorganic MNs offer a nanoscale resolution, but they are cumbersome to prepare, costly, and have low throughput. In addition, the brittle nature of silicon-based structures makes them prone to fracture, leaving residues on the skin and triggering immune reactions. These drawbacks have limited their biomedical applications (Ling and Chen, 2013). The polymeric polymer MNP in the third and fourth generations avoid these disadvantages and are the mainstream of TDDS research because of their simple preparation process, porous structure, low cost, and good biocompatibility (Liu et al., 2020c; Yang et al., 2015). Most of them are manufactured from biocompatible, biodegradable, and low toxicity materials such as hyaluronic acid (HA) (Sharma et al., 2022), alginate (Alginate) (Moniz et al., 2021), chondroitin sulfate, dextran (Dabholkar et al., 2021), chitosan (Chi et al., 2020), silk protein (Lin et al., 2021), gelatin (Dabholkar et al., 2021), polyvinylpyrrolidone (PVP) (Kim et al., 2018), polyvinyl alcohol (PVA) (Liu et al., 2020b; Zhang et al., 2020b), and polylactic acid (PLA)(Chen et al., 2015). Among them, extensive researches on the physicochemical properties of HA and its effects on the human body have proven that it is an ideal biomaterial for the medical, pharmaceutical, and cosmetic industries (Fallacara et al., 2018).

**Figure 1.**
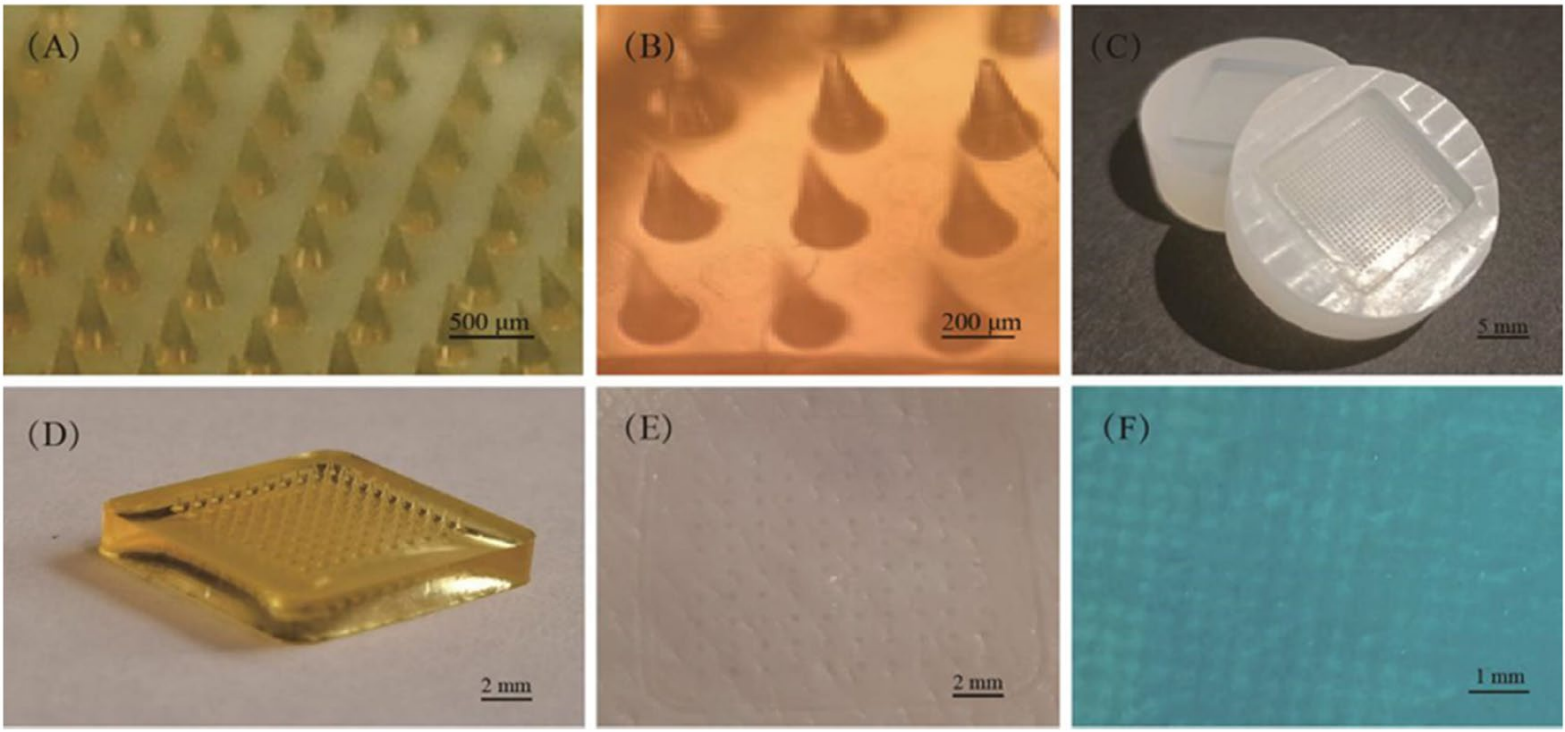
HA MNP was prepared by Huizhi Kang using the micro-molding method. (A) HA MNP (20 × 20 arrays) images photographed by a digital camera. (B) Bright-field micrograph of HA MNP (C) PDMS MNP molds (20 × 20 arrays). (D) Physical picture of HA MNP (10 × 10 arrays). (E) Mechanical strength test of HA MNP inserted into pig skin. (F) Pig skin after Trypan Blue staining.

HA dissolving microneedles patch (DMNP) was first developed by Yamamoto et al (Liu et al., 2012).The four unique properties of HA, which are human autologous source, biocompatibility, strong water locking, and viscoelastic lubricating properties (Falcone et al., 2006; Tamer, 2013), promote Hyaluronic acid microneedle patch (HA MNP) standing out among other natural polymeric materials biodegradable MNP. In this review, MNP material containing HA or cross-linked HA (x-HA) will be called HA MNP. HA MNP is relatively transparent and can maintain a certain mechanical strength on the basis of keeping a good fit with the skin (Figure 1).

Until 29 May 2022, a search on Espacenet with microneedle* as a keyword yielded 14,110 results, and a search with hyaluronic acid or hyaluronan and microneedle* found 3,165 patents filed for MNP based on HA materials, with a total share of 22.43% (https:/worldwide.espacenet.com/). Until 29 May 2022, a search on uspto.gov using microneedle* as a keyword yielded 3,824 results. A search using hyaluronic acid or hyaluronan and microneedle* as keywords found 881 patents filed for MNP based on HA material, with a total percentage of 23.04% (https://www.uspto.gov/). In addition, according to the data obtained from the ClinicalTrials.gov website, there were 70 clinical trials on MNP. Three of these were clinical studies on HA MNP (https://clinicaltrials.gov/ct2/home). So the study of HA MNP has broad research prospects and clinical value for a variety of biomedical applications.

In this review, the characteristics of HA are introduced in detail to demonstrate their excellent properties as MNP matrix. We also introduce the types of HA MNP, kinds of delivered drugs, drug distribution, application classification, and recent developments. Among them, we will focus on the biomedical applications of HA MNP in TDDS and extraction and diagnosis of biomarkers for and discuss the prospects of this field.

## 2. The introduction of the HA

**Figure 2.**
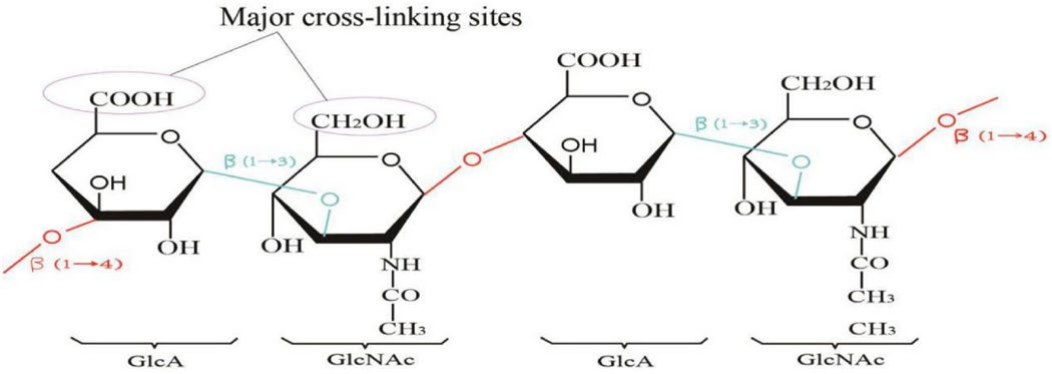
HA structure diagram and main cross-linking sites.

HA belongs to the glycosaminoglycan family (which also includes chondroitin, heparin, heparan sulfate, and keratin sulfate) and is commonly known as hyaluronan, an unbranched glycosaminoglycan composed of repeating disaccharide units of N-acetyl-D-glucosamine (GIcNAc) and D-glucuronide (GIcA) (Figure 2) (Kurisawa et al., 2005), The carboxyl and hydroxyl groups are the major cross-linking sites of HA. HA was first isolated from the vitreous humor of bovine eyes by Meyer and Palmer in 1934 (Garg and Hales, 2004). It is one of the major elements in the extracellular matrix (ECM) of vertebrate tissues. It is present in almost all body fluids and tissues, such as synovial fluid, vitreous fluid of the eye, and hyaline cartilage (Fakhari and Berkland, 2013; Necas et al., 2008). An individual with 70 kg in weight contains about 15 g of total HA with an average turnover rate of 5 g/d (Stern, 2004; Volpi et al., 2009). Fifty percent of the total HA in the human body is concentrated in the skin (Sudha and Rose, 2014), and it plays an important role in many biological processes such as cell proliferation, differentiation, morphogenesis, inflammation, and wound healing (Knopf-Marques et al., 2016; Volpi et al., 2009). Based on its biodegradability, diversity, and biocompatibility, as well as its excellent non-immunogenic properties, HA is now widely used in the treatment of various bone, eye, and skin diseases (Fakhari and Berkland, 2013).

### 2.1. Classification of HA

**Figure 3.**
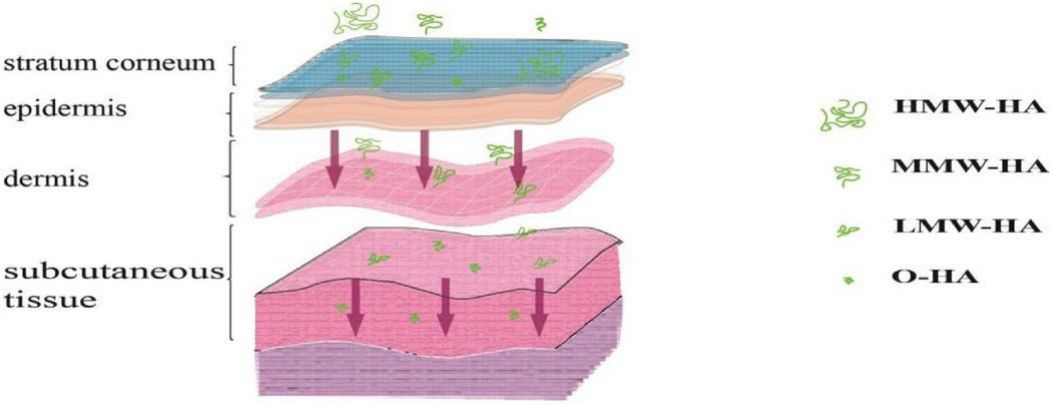
Penetration of HA in skin tissues with different molecular weights.

Based on the molecular weight of HA, HA is often classified as oligosaccharides of HA (O-HA), low molecular weight HA (LMW-HA), medium molecular weight HA (MMW-HA), high molecular weight HA (HMW-HA), and very high molecular weight HA (vHMW-HA) (Figure 3) (Tavianatou et al., 2019). Their molecular weights and actions are presented in Table 2.

**Table 2.** Molecular weights and actions of HA.

Categories	Molecular weight	Penetration	Function
vHMW-HA	>6 × 10^6^ Da	–	–
HMW-HA	>1 × 10^6^ Da	Only found in the SC of the skin	Good viscoelasticity, moisturizing, inhibition of inflammation, lubrication, and other functions.
MMW-HA	25–100 × 10^4^ Da	Can penetrate to the superficial layer	Good moisture retention, lubrication, and drug release.
LMW-HA	1–25 × 10^4^ Da	Can cross the superficial layer into the dermis	Anti-tumor, promote wound healing, promote bone growth and angiogenesis, immune regulation, and other functions.
O-HA	<1 × 10^4^ Da	Penetrate to the deeper layers of the skin	Angiogenesis, immune regulation, etc.

#### 2.1.1. Effect of molecular weight of HA on preparation and properties of MNP

In the preparation of MNP, the selection of the molecular weight of HA is particularly important. Since HA with different molecular weights has different properties (as shown in Table 2), the MNP prepared from HA have different properties in terms of mechanical strength, skin permeability, dissolution, and administration efficiency. It is worth mentioning that the molecular weight of HA has a great influence on MNP preparation. Most HA MNP are prepared from MMW-HA and LMW-HA (Chiu et al., 2018; Kim et al., 2016; Lee et al., 2014), and few are prepared from O-HA. This may be due to the moderate viscosity of MMW-HA and LMW-HA, convenient preparation of MNs, high mechanical strength, neat and complete needle shape, and superior performance. Some researchers believe that 10 kDa has good mechanical properties (Chi et al., 2022; Du et al., 2021b). In addition, HMW-HA is rarely used to prepare MNs, because the viscosity of HMW-HA is too large, and often incomplete filling occurs when filling the mold needle cavity, which affects the MNs performance (Leone et al., 2020). To comprehensively evaluate the mechanical properties of MNs in the same patch and compare the mechanical properties of different types of patches, four mechanical/material properties parameters, including displacement at rupture, rupture force, rupture stress, and Young’s modulus, were obtained to reflect the mechanical strength of MNs (Du et al., 2021b). Young’s modulus can reflect the stiffness of the measured sample, while the displacement at rupture describes the maximum deformation level of the MNs before rupture (Stubna et al., 2011). Du et al. studied two molecular weights of 10 kDa and 300 kDa HA MNs and showed that the rupture displacement of 10 kDa HA MNs (23.8 ± 7.9 μm) was significantly higher than that of 300 kDa HA MNs (12.9 ± 2.8 μm). In terms of rupture force, the average rupture force of 10 kDa HA blank MNs was 42.0 ± 9.9 mN, which was significantly higher than the 27.7 ± 5.2 mN of 300 kDa HA blank MNs. In terms of rupture stress, the blank MNs made of 10 kDa and 300 kDa HA showed similar rupture stress. The young’s modulus of the 300 kDa HA MNs was significantly higher than that of the 10 kDa HA MNs. In addition, the loading amount of drugs can significantly reduce the mechanical properties of the corresponding MNs. The mechanical strength of HA MNs decreases with the increase of molecular weight of HA, which may be due to the formation of tight molecular stacks of small molecular weight HA during MNs solidification, resulting in higher mechanical strength. In contrast, the linear structure of high molecular weight HA tends to form more turns and bends in molecular packing, resulting in inefficient molecular packing and decreased mechanical strength. Experiments by Chi et al. also confirmed that 10 kDa-HA MNs had good mechanical strength. Experiments investigated the mechanical properties of HA with three different molecular weights (10 kDa, 74 kDa, and 290 kDa) on HA MNs, using Rhodamine B as a model drug (Chi et al., 2022). The mechanical strength of 10 kDa HA MNs was found to be the highest. 74 kDa-HA MNs treatment showed the highest dose of initial delivery and longest retention time of rhodamine B in mice with a cumulative release of 96.3% at 48 h. The force required for reliable skin penetration has been reported to be 0.058 N per stitch, or 5.8 N per array (100 stitches) (Ning et al., 2020). It is worth mentioning that the mechanical strength of all types of HA MNs exceeds the minimum force required to effectively penetrate the skin. Leone et al. prepared separate MNs of 4.8 kDa, 20 kDa, 150 kDa, and 1.8 MDa and investigated the effect of four molecular weights of HA on MNP solubility in the skin, antibody response in mice, and T-cell activation *in vitro*. More importantly, Leone identified 20 kDa-HA as the best molecular weight of HA for DMNs preparation, because 20 kDa DMNs had stronger mechanical properties than 4.8 kDa and dissolved rapidly in the skin without affecting immunogenicity. The 20 kDa and 4.8 kDa HA DMNs completely dissolved within 20 min of skin use (97% ± 2% and 100% ± 0% dissolved volume, respectively), whereas the 150 kDa HA DMNs only reached 80% dissolved volume after the same use time. Leone et al. also believed that the molecular weight of HA does not affect the penetration of the MNs into the skin. The puncture efficiency of 150 kDa MNP was 96% ± 7%. The penetration efficiency of 20 kDa and 4.8 kDa HA MNs was 98% ± 4%. The low molecular weight of HA did not affect the antibody response (when below 150 kDa) or the CD4^+^-T cell response to the model antigen ovalbumin. It should be noted that environmental factors including temperature and air humidity can significantly affect the mechanical properties of HA MNs. For example, Wang et al. observed that the mechanical strength of HA MNs was significantly reduced after storage at 25 °C for 30 min at 60% relative humidity (Wang et al., 2018).

#### 2.1.2. Influence of molecular weight of HA on the biomedical application of MNP

Since different molecular weights of HA have different characteristics, MNs prepared from different molecular weights of HA have different effects in biomedical applications. From the perspective of biomedical applications of MNs, the most common of these is LMW-HA (molecular weight: 1–25 × 10^4^ Da). When HA MNP are used for the treatment of tumors and other diseases, LMW-HA is often chosen. This is because exogenous low molecular weight HA can activate Toll-like receptors (TLRs), enhance dendritic cells (DCs) antigen presentation ability, stimulate T cells to kill tumor cells, and reduce the number of tumor cells (Ghatak et al., 2002, 2005; Ward et al., 2003). The three-dimensional network structure of extracellular matrix connective tissue tends to be complete and further inhibits tumor proliferation and metastasis. The opposite HMW-HA did not have this antitumor effect. In addition, LMWA-HA can be used as an adjuvant for vaccination, so if selected on MNP materials, LMWA-HA can better activate immunocompetent cells such as macrophages (Agren et al., 1997) and DCs (Termeer et al., 2002). The presence of the opposite HMW-HA appears to reduce the immunogenicity of the antigen and is therefore not suitable as a substrate for a vaccination with MNP. However, HMW-HA is more absorbent and moisturizing, so it can be used as a substrate for wound healing MNP. In addition, when the MNP prepared by LMW-HA are inserted into the skin, the hyaluronidases (HYALs) in the body degrade the MNP very quickly to monosaccharides, which facilitates the release of the encapsulated drugs. In addition, because MMW-HA and LMW-HA have moderate molecular weight, they are suitable to be modified on their linear polysaccharide structures to form copolymers or vesicle structures to wrap drugs and loaded in HA MNP matrix to meet their therapeutic needs. MMW-HA and LMW-HA are also conducive to covalent binding with therapeutic drugs and specifically targeted accumulation of drugs to tumor sites. In conclusion, the difference in molecular weight of HA leads to various biological applications. Table 3 lists several representative examples to illustrate the impact of MNP with different molecular weights of HA in biomedical applications.

**Table 3.** Effects of different molecular weight HA MNP in biomedical applications.

Classification	Common characteristics	Example	Application	Advantages	Reference
HMW-HA >1 × 10^6^ Da	Strongest water absorption and good moisture retention	1500 kDa	Wound Healing	Absorption of tissue exudate Synergistic effect on wound healingl	Yao et al., 2021
MMW-HA 25–100 × 10^4^ Da	Facilitate the formation of vesicles	300 kDa	Diabetes	Form hypoxic sensitive hyaluronic acid (HS-HA) vesicles	Yu et al., 2015
LMW-HA 1–25 × 10^4^ Da	Good mechanical properties	60 kDa	Extraction and Diagnosis	Good water absorption Easy for chemical modification The receptors cluster of differentiation-44 (CD44) targeting	Puigmal et al., 2021
		40 kDa	Breast Cancer	Form amphiphilic conjugated polymers (HA − GMS) CD44 targeting	Sharma et al., 2022
		30-100 kDa	Psoriatic arthritis	Have synergistic effect on inflammation Stable drug release.	Yu et al., 2021a
		20 kDa	Immunotherapy	TLRs targeting Enhance immune function	Kim et al., 2019
		10 kDa	Superficial Tumors	Provides acidic and anaerobic environments to maintain the activity of 5-Ala CD44 targetsing	Zhu et al., 2019
O-HA <1 × 10^4^ Da	Expensive price	4.8 kDa	—	—	Leone et al., 2020

### 2.2. HA receptors

The discovery of HA receptors has made a quantum leap in the understanding of HA, revealing its important role in embryonic development, tumor invasion, and tissue healing by binding to receptors and regulating cellular functions. These include CD44 (Underhill, 1992), TLRs, RHAMM (receptor for HA-mediated motility), and LYVE-1 (lymphatic vessel endothelial hyaluronan receptor-1), and hyaluronan receptor for endocytosis (HARE).

#### 2.2.1. TLRs

**Figure 4.**
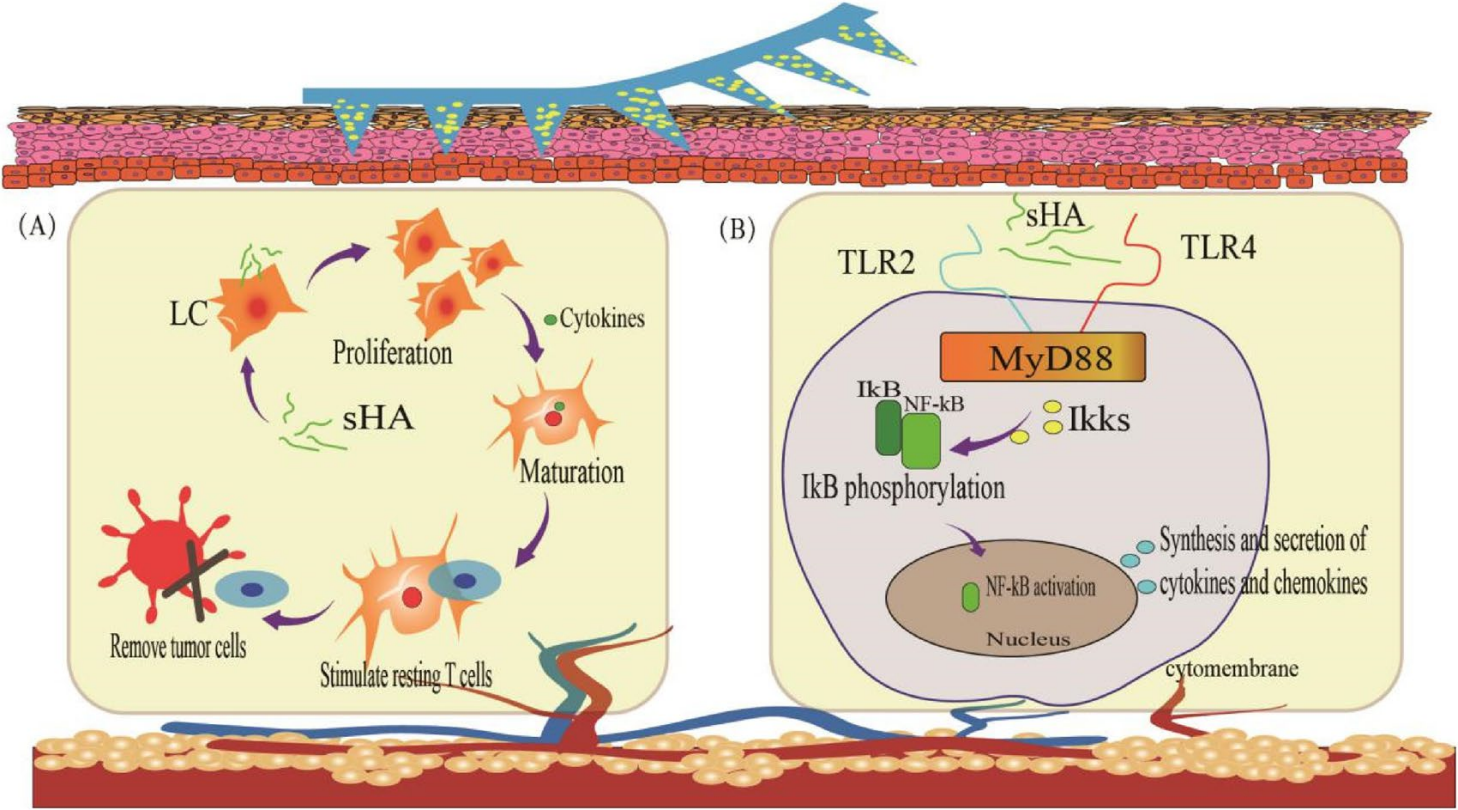
Immunity in the skin with sHA. (A) sHA induces LCs migration, proliferation, and maturation. (B) HA activates the MyD88-dependent TLRs signaling pathway.

TLRs belong to the family of pattern recognition receptors (PRRs) that recognize highly conserved microbial component-pathogen-associated molecular patterns (Takeda and Akira, 2007). TLRs are mainly expressed in immune cells, including macrophages, DCs, B cells, and T cells. To date, 10 TLR genes have been identified in humans. Certain types of HA could induce TLR based inflammation. To investigate the potential role of TLRs in mediating HA signaling during inflammation, Jiang et al. (Jiang et al., 2005) used peritoneal macrophages from myeloid differentiation factor 88 (MyD88)-, TLR1-, TLR2-, TLR3-, TLR4-, TLR5- or TLR9-deficient mice. In MyD88-deficient macrophages, stimulation of chemokine gene expression by HA fragments was abrogated. Chemokine macrophage inflammatory protein 2 (MIP2) expression was reduced but was still present in TLR2- and TLR4-deficient macrophages. It is inferred that HA signaling may require TLR2 and TLR4. Since this review only discusses HA MNP, we only briefly summarizes the immunization of HA degradation products, fragments of tetrasaccharide or hexasaccharide size (sHA) (Termeer et al., 2000). And sHA binds to the intracellular connector protein MyD88 via TLR2 and TLR4, which interacts with human interleukin-receptor-associated kinase (IRAK) to mediate TLR-induced signal transduction (Figure 4). Activation of TLRs signaling pathway mainly causes the activation of nuclear transcription factor (NF-κB). After entering the nucleus, NF-κB induces the secretion of inflammatory cytokines such as IL-1β, IL-6 and tumor necrosis factor-α (TNF-α) and the expression of type I interferon, which is involved in inflammatory and immune responses. It is worth mentioning that sHA-related immune responses are also associated with Langerhans cells (LCs). DCs in the skin are called LCs, which are essential for the initiation of immune responses in the skin. When activated, LCs move to the lymphoid tissue to interact with T and B cells to stimulate and control appropriate immune responses. HA induces LCs to migrate, proliferate, and mature. Activation of resting T lymphocytes through the dermis into local lymph nodes enhances the body’s immune response and thus clears tumor cells.

#### 2.2.2. CD44

CD44 contains a HA binding domain or chondroitin domain, a membrane-proximal structural domain, and a cytoplasmic tail. It is an abundant and functionally important receptor expressed in a variety of cell types such as leukocytes, fibroblasts, epithelial cells, keratinocytes, and some endothelial cells (Misra et al., 2011). A large number of HA receptors, CD44, exist on the surface of certain superficial epidermal solid tumors. HA is used as a targeting carrier for anti-tumor drugs, and using HA MNP can wrap drug molecules in the three-dimensional structure of the HA MNP matrix. With the degradation of the needle body, the drugs which adhered to HA release, and tumor cell surface receptors targeted binding to increase the absorption and retention time of the drug in the tumor. Thus, the efficacy of the drug is improved and toxic side effects are reduced.

**Figure 5.**
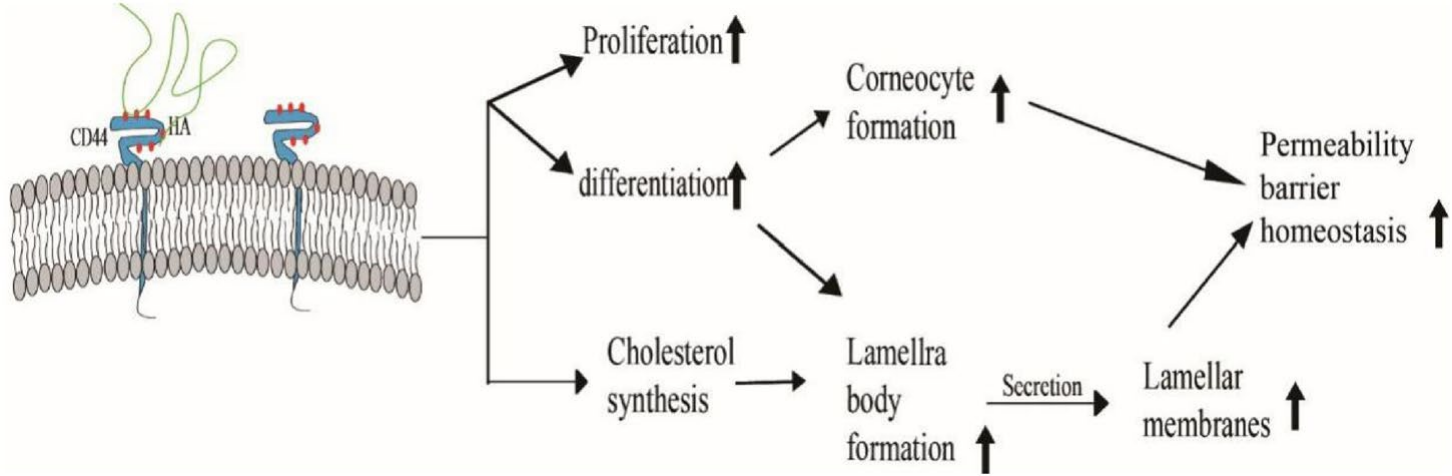
Effect of HA on epidermal structure and function after interaction with CD44.

In addition, HA-CD44 interaction (Figure 5) also affects epidermal structure and function by upregulating the expression of differentiation markers (e.g., involucrin [IVL], profilaggrin, and keratin 10 [K-10]) to trigger proliferation and differentiation of keratin-forming cells and regulating lipid synthesis or secretion, thereby affecting the homeostasis of the permeability barrier (Bourguignon et al., 2006).

### 2.3. Regulatory pathways of HA

HA is associated with double-sided properties in several regulatory processes. The double-sided properties are that HA with different molecular weights has opposite properties in anti-inflammatory, oxidation, angiogenesis, and tumor. Except for molecular weight, the double-sided properties are related to cell-specific (Cyphert et al., 2015).

#### 2.3.1. HA and inflammation

When HA MNP penetrate the skin, HA trigger an immune response in the skin. The effect of HA in immune response is subject to the molecular weight. LMW-HA shows pro-inflammatory properties. Several studies have identified TLR4 and TLR2 signaling as key players in the pro-inflammatory properties of LMW-HA (Campo et al., 2012; Campo et al., 2013; D’Ascola et al., 2020; Jiang et al., 2005). For example, activation of murine and human alveolar macrophages, or induction of irreversible phenotypic and functional maturation of human DCs (Liang et al., 2011; McKee et al., 1996). LMW-HA can bind to TLRs, leading to the production of pro-inflammatory cytokines and chemokines in various cell types. However, not all HA with small molecular weights have pro-inflammatory properties, and some studies have shown anti-inflammatory of O-HA, such as reducing poly(I: C)/TLR3-induced inflammation and inhibiting the release of IL-6 and TNF-α (Kim et al., 2013). Therefore O-HA can act directly on lymphocytes to modulate the onset of inflammatory demyelinating disease (Winkler et al., 2013). HMW-HA shows anti-inflammatory and immunosuppressive properties (Cyphert et al., 2015). Campo et al. suggest a mechanism: inhibition of cell surface receptor-ligand interactions by wrapping a mucoadhesive protective layer around the cell surface. HMW-HA plays a protective role in inflammatory pathologies, such as rheumatoid arthritis (Campo et al., 2012).

#### 2.3.2. HA and oxidation

HA has antioxidant activity (Campo et al., 2004; Kim et al., 2008). The different antioxidant effects of HMW-HA include neutralization of reactive oxygen species (ROS) outside polymorphonuclear leukocytes while protection of neighboring cells (Moseley et al., 2003), reduction of UV-induced cell damage (Li et al., 2013), reduction of ethylenediaminetetraacetic acid (EDTA) induced oxidative DNA damage (Ye et al., 2012), and protection against ROS-induced cellular repair of ischemia-reperfusion injury in cardiac myocytes, etc. Oxidative stress is caused by an imbalance between the amount of ROS and antioxidant capacity. It is speculated that especially the HMW-HA can protect cells from ROS, and the HMW-HA with hydroxyl functional groups may absorb ROS (Onodera et al., 2015). The mechanism may be related to HA degradation, CD44 receptors, a complex combination of effects, and the specific mechanism of antioxidant effect is still unknown and needs further study. In addition, HMW-HA forms a cytoprotective layer on the cell membrane, thus protecting cells from apoptosis. The antioxidant function of HMW-HA helps to mitigate DNA damage in human leukocytes during oxidative stress. As mentioned earlier, most HA biological effects are closely related to their molecular weight.

#### 2.3.3. HA and angiogenesis

Both HMW-HA and LMW-HA are effective regulators of angiogenesis, but they have opposite effects on endothelial cells (ECs) proliferation and motility (Litwiniuk et al., 2016). LMW-HA has been shown to stimulate vascular ECs proliferation, migration, and tubule formation *in vitro* and in various angiogenic models *in vivo*. While HMW-HA showed anti-angiogenic properties by inhibiting ECs proliferation, motility, and tubule formation (Cyphert et al., 2015; Girish and Kemparaju, 2007; Jiang et al., 2011). Although LMW-HA has the ability to promote angiogenesis, O-HA differs. In several types of tumor xenografts, injection of O-HA inhibits rather than stimulates tumor growth. This may indicate up-down correlation responses to different forms of HA and the possible role of the microenvironment in this process. Rooney et al. demonstrates a relationship between angiogenic O-HA and the production of collagens during the process of angiogenesis *in vivo* and *in vitro* (Rooney et al., 1993).

#### 2.3.4. HA and tumor

Exogenous LMW-HA has an antitumor effect. Ghatak et al. found that exogenous LMW-HA can inhibit the growth of mouse TA3/ST breast cancer cells, rat C6 glioma cells, HCT 116 human colon carcinoma cells, and human lung cancer cells *in vitro*, with an inhibition rate of 50%–100%. The mechanism may be related to the competitive binding of CD44 receptors (Ghatak et al., 2002, 2005; Lesley et al., 1993; Ward et al., 2003). Further studies showed that exogenous LMW-HA inhibited the growth of different types of tumor cells by inhibiting phosphatidylinol 3-kinase (PI3K) activity and serine/threonine-protein kinase (SIK2) phosphorylation. And exogenous LMW-HA can activate TLRs, strengthen the DCs antigen-presenting ability, stimulate T cells to kill tumor cells, reduce the number of tumor cells, Hexosidase hydrolysis ability is reduced, the extracellular matrix of connective tissue three-dimensional mesh structure tends to be complete, further inhibiting tumor proliferation and metastasis. Because of these unique properties of HA, HA MNP is widely used in tumors, diabetes, psoriasis, arthritis, and other diseases. HA material can play a role in both medicine and adjuvant. We will further elaborate in 7.1. in combination with HA MNP studies in recent years.

### 2.4. HA degradation

The human body itself continuously synthesizes and degrades natural HA. The degradation of HA can be regarded as a depolymerization process mediated by the cleavage of the glycosidic bond (the hexosaminidic β (1 → 4) linkages between N-acetyl-D-glucosamine and D-glucuronic acid residues) (Jiang et al., 2007). And the degradation of HA in humans involves two main mechanisms: specific enzymatic degradation and nonspecific free radical degradation. For enzymatic degradation, there are six known human HYALs: HYALs1-4, PH-20, and HYALP1 (Jiang et al., 2007). HYAL2 degrades the high molecular weight HA (>1 MDa) to a relatively low molecular weight (∼20 kDa). After this process, HYAL1 degrades the low molecular weight HA to tetrasaccharide. Finally, the tetrasaccharide is degraded to monosaccharide by D-glucuronidase, and N-acetyl-D-glucosaminidase (Csoka et al., 2001). In addition, polymers with molecular weights below 40 kDa can be cleared from the body by renal excretion. Their degradation residues are very safe. For free radical degradation, HA can react with free radicals to eliminate them while being degraded. HYAL and ROS together partially degrade about 30% of the total amount of 15 g HA in the body. The remaining 70% is systematically catabolized: HA is mainly transferred via the lymphatic circulation to the lymph nodes, where it is internalized and broken down by the endothelial cells of the lymphatic vessels (Fallacara et al., 2018). In addition, a small fraction of HA is taken into the bloodstream and degraded by hepatic endothelial cells (Heldin et al., 2019), and can also be metabolized by granulocytes or by ROS released from UV-irradiated skin (Fraser et al., 1997).

## 3. Characteristics of HA as a MNP material

HA and its cross-linked derivatives are often used as MNP materials. Both HA derivatives degradation and HA degradation are both completely harmless to humans. Compared to natural HA, x-HA derivatives have significantly improved physical and chemical properties, without losing their inherent advantages such as biocompatibility and biodegradability.

### 3.1. Drug delivery

In terms of transdermal drug delivery, scientists have made several attempts to develop biosafety HA MNP, which no longer simply acts locally to replace traditional ointments and patches, but aims to deliver biological macromolecules such as proteins, vaccines, and nucleic acids. In addition to traditional percutaneous administration, MNP can replace low-dose injections and improve patient compliance. The advantage of HA MNP over other MNP is that they contain a large number of natural polymers with carboxyl groups, and HA MNP provides an acidic, anhydrous, and oxygen-free environment for drugs, which can inhibit the degradation and dimerization of certain acidic drugs. In addition, HA MNP has the ability to encapsulate proteins, enzymes and antibodies in a polymer matrix. At the same time, it can maintain the biological activity of certain peptides and proteins (Du et al., 2021a; Fonseca et al., 2019). It also allows for a sustained and relatively rapid release of enzymes (Di Natale et al., 2021; Panda et al., 2021) and antibodies (Monkare et al., 2015).

### 3.2. Treatment and extraction diagnostics

In the treatment of diseases, HA MNP is easily degraded after insertion into the skin, facilitating rapid transdermal delivery of anti-inflammatory (Diclofenac, Melittin, etc.) and analgesic drug loading. In addition, HA MNP can also be used for skin cancer treatment because of the interaction between HA and CD44 receptors, and the overexpression of HYALs in the tumor microenvironment can help HA to enzymatically dissolve at the tumor site and activate drug release. As well, it can play a targeting role and increase the efficacy of the drug. Similarly, the CD44 receptor is also highly expressed in psoriasis, and it was found that HA can achieve an anti-allergic effect by inhibiting the interaction between CD44 and PKCa. Therefore, HA MNP is preferred when designing treatments for diseases such as skin cancer and psoriasis. More broadly, because HA is not only an endogenous substance in the skin’s surface, but also in dermis with the ability to retain cellular moisture and improve lesion healing, it can also significantly accelerate keratin-forming cell proliferation/migration and angiogenesis by inducing the production of vascular endothelial growth factor (VEGF) and adhesion molecules. Therefore, it is useful for wound healing and skin care in any situation. Secondly, in terms of extraction diagnostics, HA MNP is overwhelmingly superior in terms of their use for diagnostic extraction. As HA can absorb water hundreds to thousands of times heavier its own weight, the natural water absorption capacity of HA (Papakonstantinou et al., 2012) makes it an ideal candidate for rapid interstitial skin fluid (ISF) extraction. The x-HA not only retains its water absorption capacity but also increases its mechanical strength. In addition, the hydrogel MNs made of x-HA can extract ISF quickly and in one step in a short time.

### 3.3. Formulation design

For MNP formulation design, we can also take advantage of the easy degradation of HA and combine it with other materials with different solubility for rational drug design. For example, Chiu et al. (Chiu et al., 2018) developed a composite MNP for biphasic antigen release consisting of three components: HA at the tip (rapid release of antigen), chitosan in the middle (slow release of antigen), and PVP/PVA at the base (supportive effect). The biphasic release system (combination of rapid release and sustained release) is more conducive to stimulating antibody production than the conventional sustained release system. Moreover, HA has many modification sites. Different HA modification methods and cross-linking agents can be selected freely depending on the therapeutic target. For example, the rate of drug release and mechanical strength can be controlled by adjusting the cross-linking density of chemical cross-linking sites. HA and x-HA can also be used in combination to achieve similar effects when designing MNP formulations.

## 4. Types of HA MNP

The mechanical strength, insertion depth, and drug release profile of MNP is regulated by adjusting and changing the matrix composition and degree of cross-linking of HA MNP to achieve rapid, slow, or stimulus-responsive drug release. In this review, HA MNP is classified into three types rapid drug-releasing HA MNP (RDR HA MNP), sustained drug -releasing HA MNP (SDR HA MNP), and stimuli-responsive HA MNP (SR HA MNP) according to the different drug release behaviors (Table 4).

**Table 4.** Three types of HA MNP with different drug-release behaviors.

	RDR HAMNP	SDR HAMNP	SR HAMNP
Material	Pure HA, HA and other polymers	X-HA	Modified HA, x-HA
Drug release properties	Fast	Slow	Depending on the response conditions
Mechanical strength	Weak	Strong	Strong
Loading capacity	Limited drug loading capacity (mainly concentrated in needle tip)	Large drug loading capacity (needle tip or substrate can be loaded)	Depends on the design
Advantage	The rapid release of the drug produces a curative effect	It has the effect of sustained and controlled release	Potential for automated on-demand drug delivery (diabetes)

### 4.1. Rapid drug-releasing HA MNP

**Figure 6.**
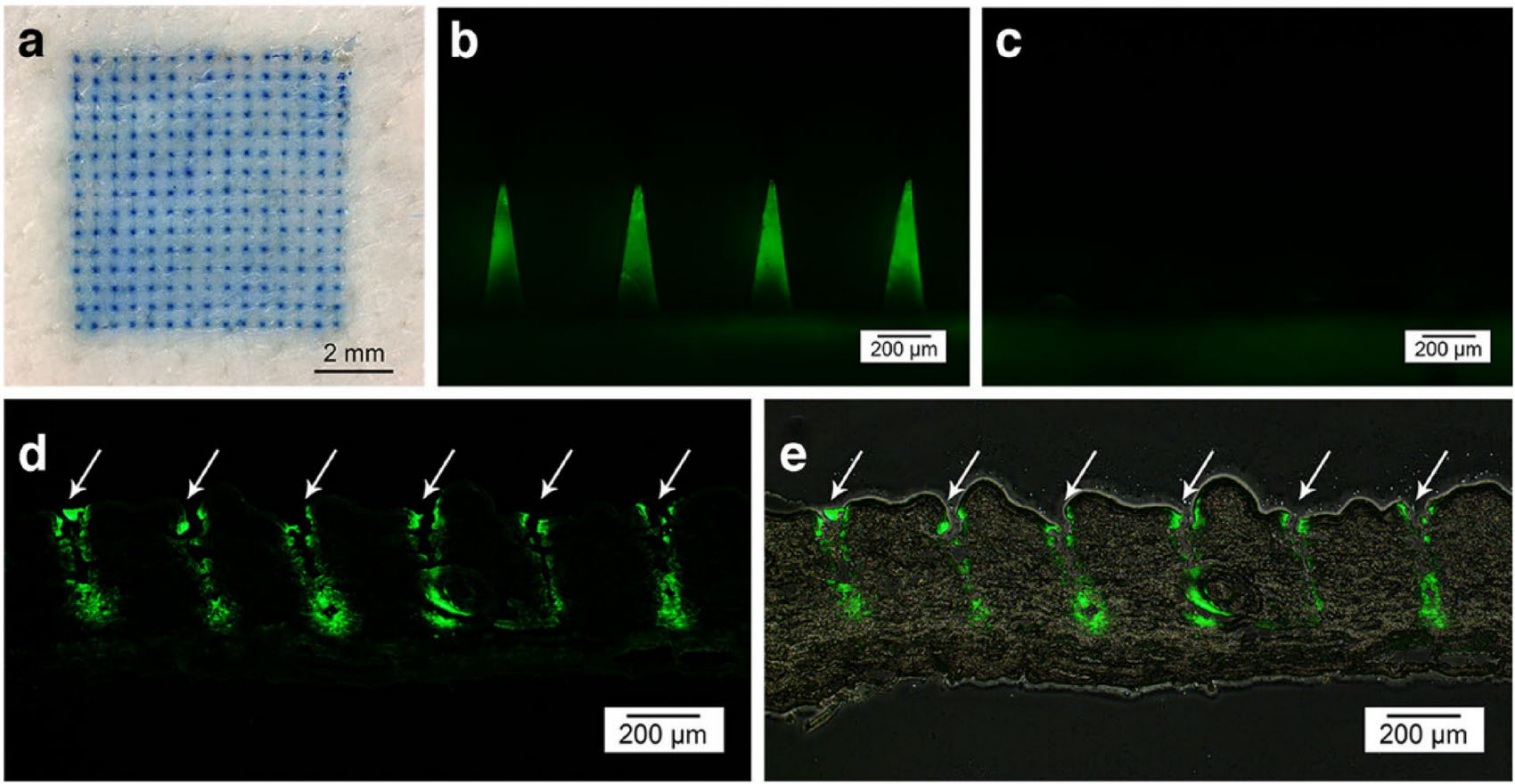
Fluorescence micrographs of HA MNP loaded with carboxyfluorescein-labeled EXT after skin insertion. (a) Bright-field micrographs of neonatal pig skin after *in vitro* insertion of methylene blue-loaded MNP. (b) Skin before and (c) after 2 min of insertion. (d) Fluorescence of histological sections of pig skin puncture sites (white arrows) and (e) combined bright-field and fluorescence images. Copyright 2014, Springer.

RDR HA MNP is prepared from HA, or a mixture of HA and other polymers (PVP, PVA, CMS, etc.). Because of its ability of rapid dissolving in human body and thus achieve rapid drug release, the MNP is inserted into the skin where HA quickly degrades in the skin extracellular fluid, releasing the drug. Zhu et al. reported HA MNP containing exenatide (EXT) drug at the tip of the needle (Figure 6). *In vitro*, drug release results showed that the release rate of the loaded drug was about 80% after 30 s of MNP insertion into the skin, and almost all of it was released within 2 min. The results of *in vivo* animal experiments showed that the therapeutic effect of the MNP for type 2 diabetes was comparable to that of conventional subcutaneous insulin injection, with a relative bioavailability of 97%. A fast-acting therapy was achieved (Zhu et al., 2014). In addition, HA is a natural polymer containing a large number of carboxyl groups, and HA MNP provides an acidic, relatively anhydrous, oxygen-free environment for drugs that inhibits degradation and dimerization of certain drugs. For example, Vitamin C (Vc) is a substance that tends to degrade during storage. Wang et al. used a HA DMNP loaded with arbutin and Vc to inhibit UVB-induced skin pigmentation. The results showed that the stability of Vc in DMNP was better than that in emulsions, whether stored under shade conditions or in light (Wang et al., 2021). HA DMNP provide an acidic anaerobic environment for 5-ALA and reduce the dimerization of 5-ALA molecules through Schiff base bonding to form inactive pyrazine derivatives, thus maintaining their chemical structure and biological activity (Zhu et al., 2019).

### 4.2. Sustained drug -releasing HA MNP

SDR HA MNP is generally prepared by x-HA. The main function of the cross-linking agent is to link the ‘strip’ HA molecular structure one after another. As mentioned earlier, HA is prone to degradation and poor mechanical strength *in vivo*. The mechanical strength, stability, and anti-degradation properties of MNP is enhanced by modifying HA to maintain it *in vivo* for a longer period time, resulting in a series of novel bioactive HA-derived MNP that have expanded their application in biomedical and tissue engineering fields. Compared with natural HA, x-HA have significantly improved physical and chemical properties, but their biocompatibility and biodegradability remain unchanged. The rate of drug release can be controlled by adjusting the cross-linking density at the physical or chemical cross-linking sites. It has been demonstrated in the literature that the diffusion coefficient of the drug is found to be 6.45 times lower than that of the non-crosslinked system when the cross-linking degree reaches 90%. The main reason for the decrease in drug diffusion ability is that the cross-linking reaction changes the conformation of the polymer. A larger degree of cross-linking makes the polymer coils more compact and close to each other, eventually forming a continuously distributed cross-linked network, which decreases its degradation rate *in vivo*. At the same time, these cross-linked networks also impede the interaction of soluble drugs with tissue fluids, thus preventing premature drug release (Feng et al., 2021). The HA chains can be modified with hydroxyl, carboxyl (He et al., 2013), and acetylamino groups, among which the modification of hydroxyl and carboxyl groups is the current hot topic. Commonly used crosslinkers for HA MNP preparation are methacrylic anhydride (MA), 1,4-butanediol diglycidyl ether (BDDE), NHS-terminated 8-arm PEG, Gantrez S-97 (GAN).

MeHA is highly hydrophilic and can swell rapidly within minutes. A swelling rate of approximately 600% can be achieved *in vitro* in 30 seconds (Park et al., 2022). Therefore, in addition to transdermal drug delivery, it can also be applied to the immediate extraction of biomolecules from tissue fluids for analysis, such as glucose and cholesterol (Chang et al., 2017). The swelling rate can be adjusted by adding a suitable photo-initiator to control the UV exposure time. Cross-linking time was inversely proportional to swelling rate and drug-carrying capacity (Chew et al., 2020). MeHA not only enhances the mechanical strength of MNP and extends the duration of skin microchannels, but also produces a longer peak time of drug action T_max_ and a higher peak concentration of drug C_max_. The degree of methacrylate can be adjusted according to treatment needs. The degree of cross-linking has a great impact on the mechanical strength of MNP. MNP varies greatly in morphology and properties depending on the concentration of MeHA (Yao et al., 2021), and this review lists several different MeHA-prepared HA MNP. HA MNP prepared with MeHA can be used for diagnostic extraction and transdermal delivery (Table 5).

**Table 5.** Several HA MNP were prepared from MeHA.

Objective	Experiment procedure	Purpose	Conclusion	Reference
Analysis	Synthesis and fabrication of MeHA MNP Effect of Crosslinking Sequence on Swelling Behavior of MeHA MNP Cyclic Voltammetric Analysis of Model Biomolecules Effect of MN Swelling Time on Biomolecules Measurement Measurement of Biomolecules in a Gelatin Phantom and Porcine Skin	Electrochemical Analysis	Achieve fast extraction and measurement of a biomolecule in the ISF	Park et al., 2022
Extraction	Preparation and characterization of MeHA The mechanical strength of cross-linked MeHA-MNP The swelling abilities of cross-linked MeHA-MN patches The extraction of ISF *In vivo* performance of the MNP	Extract glucose and cholesterol	Extract sufficient ISF in a few minutes	Chang et al., 2017
Drug delivery	Development and characterization of swellable MeHA MNP Five-minute UV exposures yield MN structures CL5-MeHA MNP of drug loading Mechanical property and drug release profile of CL5-MeHA MNP Adhesion strength of CL5-MeHA MNP	Delivery of fluorescein, FITC-Dextran adriamycin (DOX)	Drug-loading via the swelling effect of a hydrogel MN patch	Chew et al., 2020
	Synthesis of MeHA Fabrication and characterization of MNP Evaluation of cytocompatibility Drug release property of DOX/MNP *In vivo* transdermal drug delivery	Delivery of DOX	Compared with DMNP, SMNP can significantly improve transdermal drug delivery efficacy	Yu et al., 2021b

BDDE is the most commonly used cross-linking agent in the preparation of cosmetic HA fillers. BDDE reacts mainly with the primary alcohol in the HA backbone. As the glycosidic bonds in the polysaccharide backbone are maintained after the reaction, the x-HA is as susceptible to *in vivo* degradation mechanisms. BDDE is biodegradable and the higher the amount of BDDE, the higher the degree of cross-linking of HA. The degree of cross-linking or modification can be confirmed by 1H-NMR studies, but its dosage should not be too high. It is less toxic compared to other ether-bonded cross-linking agents, and the residual content of cross-linking agent in it can be detected by fluorescence detection. HA MNP cross-linked by BDDE can delay the degradation of HA and achieve a slow-release drug effect. For example, Choi et al. compared the penetration success, mechanical strength, and dissolution rate of pure HA MNP and x-HA MNP. The latter was found to degrade more slowly than the former, but completely within 200 hours (Choi et al., 2018b). And the addition of cross-linking agent resulted in a strong elastic behavior of the skin after drug administration. In addition, this behavior also helps protect the MNP tip from the impact of administration. Zhang et al. used MNP prepared by cross-linking HA with BDDE as anti-aging therapy and *in vitro* and *in vivo* degradation tests confirmed the stability and effectiveness of the polymer matrix over a long period of time (Zhang et al., 2018).

GAN is also a common cross-linking agent. Larraneta et al. described the preparation of HA hydrogel MNP crosslinked with GAN. The esterification reaction between the acid group of GAN and the polyol group of HA, in contrast to the use of organic solvents or potentially toxic reagents, requires potential advantages over other synthetic methods (Larraneta et al., 2018). In addition, Puigmal et al. demonstrated the reaction of HA primary amines with 8-arm PEG-NHS containing succinimide-functional groups and experimentally found that the type of cross-linker had a significant effect on the swelling rate of hydrogel MNP. By fine-tuning the molar mass and relative ratio of the crosslinker, the mechanical strength of MNs could be effectively improved and irreversible crosslinking strategies were avoided (Puigmal et al., 2021).

### 4.3. Stimulus-responsive MNP

**Figure 7.**
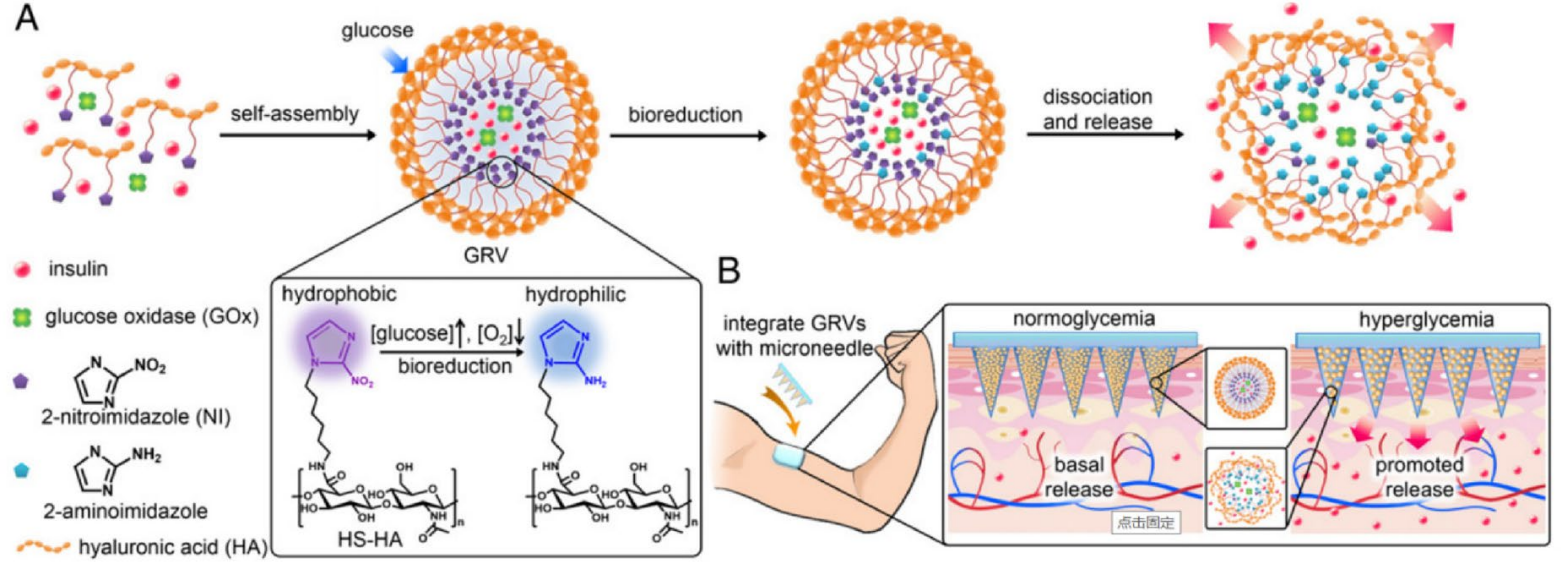
Schematic of the glucose-responsive insulin delivery system using hypoxia-sensitive vesicle-loading MNP. (A) Formation and mechanism of GRVs composed of HS-HA. (B) Schematic of the GRV-containing MNP (smart insulin patch) for *in vivo* insulin delivery triggered by a hyperglycemic state to release more insulin. Copyright 2015, National Academy of Sciences.

The construction of stimulus-responsive drug delivery systems is one of the hot research topics in the field of drug delivery (Cahill and O’Cearbhaill, 2015). Stimulation by physiological conditions (pH, glucose concentration, enzyme activity, etc.) or external environment (sound, light, current, magnetic field, temperature, bacterial strain, etc.) is the trigger for MNP drug release (Gowda et al., 2022). Polymeric MNP with stimulus-responsive properties holds promise for on-demand drug delivery and better therapeutic efficacy. Stimulus-responsive MNP is commonly made of x-HA. X-HA can provide a stable MNP internal environment, reduce drug loss and modulate mechanical strength to meet the needs of different stimulus-responsive drug delivery systems. For example, Yu and coworkers developed a novel glucose-responsive MNP smart patch (Yu et al., 2015). The entire MNP matrix is made of MeHA to improve the stiffness of MNP and limit the loss of glucose-responsive vesicles (GRVs) within the needle. GRVs are self-assembled from amphiphilic hypoxia-sensitive HA (HS-HA) with a core encapsulating recombinant human insulin and glucose oxidase (GOx) (Figure 7). HS-HA was synthesized from hydrophobic 2-nitroimidazole (NI) moiety and hydrophilic HA, which was used in order to achieve rapid hypoxic response transduction of vesicles, which are very sensitive to hypoxic conditions at the tumor site. Under hypoxic conditions, the hydrophobic component NI can be converted to hydrophilic 2-aminoimidazole by a series of one-electron reduction reactions with nitroreductases coupled to biological reducing agents (e.g., NADPH, an abundant coenzyme in tissues). The local hypoxic microenvironment induced by Gox oxidation in the hyperglycemic state promotes the reduction of HS-HA, which rapidly triggers vesicle dissociation and consequently the release of insulin, effectively regulating blood glucose concentrations. MNs treated with GRV containing insulin and enzymes rapidly reduced blood glucose to nearly 200 mg/dL within 0.5 h and maintained normal blood glucose status within 4 h (<200 mg/dL).

In 2016, Ye et al. again utilized the strategy of Gox-induced local hypoxia to promote HS-HA vesicle dissociation (Yu et al., 2015). Unlike before, they utilized glucose to promote insulin secretion through diffusion interacting with pancreatic β-cells loaded in MNP to treat type 1 diabetes. This strategy in conjunction with a glucose signal amplifier (GSA) triggers insulin secretion in ß-cells capsules. Yu et al. (Yu et al., 2017) conducted another study in which glucose-responsive vesicles were loaded in an x-HA MNP matrix. Vesicles are self-assembled from diblock copolymers that are hypoxic and sensitive to H_2_O_2_. To effectively regulate blood glucose at 10 h in chemically induced type 1 diabetic mice. This shows that x-HA is a reliable MNP matrix for loading GRVs, insulin, and pancreatic β-cells.

The development of intelligent and portable HA MNP has been the general trend. SR MNP, diagnostic and therapeutic integrated MNP and wearable MNP will become hot spots in the MNP research field. In terms of existing research, the vast majority of HA MNP TDDS is concerned with the development of SR HA MNP based on physiological conditions or external conditions. Only limited reviews focus on the combination of MNP with electrochemical sensors and chips to achieve closed-loop control of drug release. We imagine that HA MNP can also be designed to not only administer drugs on demand but also to personalize the design to improve the quality of life of patients.

## 5. Delivery of substances

The SC is considered to be a great obstacle to the effective delivery of drugs and cosmeceuticals through the skin. The ideal molecular properties for a well-penetrated SC are molecular weight <500 Da, log P between 0-3, and the hydrogen bond acceptor or donor in the molecule is less than 2. (Brown et al., 2006). MNP can physically create a micro-channel on the skin surface to deliver drug molecules to the dermis.

Unlike MNP made of other materials, HA is a natural polymer containing a large number of carboxyl groups, and HA MNP provides an acidic, relatively anhydrous, and oxygen-free environment for drugs, which can inhibit the degradation and dimerization of certain drugs, so when delivering acidic drugs, HA can be chosen as the MNP material in the first place. In addition. HA MNP has the ability and potential to encapsulate proteins, enzymes, and antibodies within a polymeric matrix, maintaining the bioactive structure of certain peptides and proteins (Panda et al., 2021). For example, MeHA can stabilize peptides and HA can stabilize lysozyme. HA MNP can also achieve sustained yet relatively rapid release of enzymes, and antibodies and avoid aggressive gastrointestinal tract or blood environment. Therefore, HA MNP can be given priority when delivering proteins, enzymes, and antibodies. In addition, hormonal drugs can remain highly stable in MNP without causing serious skin damage. For example, Macedo et al. showed that novel insulin-loaded MNP prepared from HA is a very useful alternative, showing that insulin-loaded MNP is highly stable when stored at −40, 4, 20, and 40 °C for one month of insulin. Transdermal delivery of drug molecules is not limited to less than 500 Da due to the use of MNP. HA MNP has been found to enhance the permeability of drug small molecules such as 5-ALA, amphotericin, caffeine, curcumin (Cur), paclitaxel, adriamycin, and ivermectin (IVM), which represent hydrophilic small molecules <500 Da (5-ALA 131.13 Da, caffeine 194.19 Da, amphotericin 325.42 Da), hydrophilic molecules (>500 Da) (Adriamycin 543.52 Da). There are also hydrophobic small molecules <500 Da (picrotoxin 288.31 Da, Cur 368.39 Da) and hydrophobic macromolecules (>500 Da) (IVM 875.09 Da). Notably, in addition to these unique advantages, HA MNP can deliver most of the other drugs that MNP can deliver, including small molecules, biomolecules of other compounds that currently lack oral formulations (proteins, hormones, vaccines, RNA, etc.), other substances such as complex vesicles and encapsulated solid nanoliposomes. Tables 6-10 list HA MNP delivery of small molecule drugs, protein drugs, hormonal drugs, vaccines, and genetic materials, respectively. The MNP matrices in the following table are all HA or x-HA.

**Table 6.** Summary of HA MNP delivery of small molecule drugs.

Drug	Limitation	Adaptation disease	Results	Reference
5-ALA	Hydrophilicity, zwitterionic nature limit its penetration	Skin lesions	Enhance 5-ALA penetration and produce protoporphyrin IX (PpIX) in deeper skin lesions.	Champeau et al., 2020
Amifostine	Side effects (e.g., hypotension, nausea, and emesis)	Hematopoietic injury induced by ionizing radiation.	Long-term protection of the hematopoietic system from radiation within 3-7 h pre-radiation	Yu et al., 2020
IVM	Low oral bioavailability	Parasitic diseases	Bioavailability has improved *In vitro* and *in vivo* release assays showed slow and sustained release for up to 9 days	Chen et al., 2019
Caffeine	Low bioavailability	Obesity	Bioavailability has improved The weight of high-fat diet-induced obese mice lost about 12.8% ± 0.75% after administration for 6 weeks	Dangol et al., 2017
Shikonin	Low bioavailability	Hypertrophic scars	Bioavailability has improved Reduce the viability and proliferation of the hypertrophic scar derived fibroblasts (HSFs) and downregulate the fibrotic-related genes	Ning et al., 2021
Cur	Low solubility, poor stability, and systemic bioavailability	Melanoma	The MNP delivered 74.7% of their drug load over 6 h	Cheng et al., 2020

**Table 7.** Summary of HA MNP delivery of protein drugs.

Therapeutic agent	Advantage	Research purpose	Results	Reference
Melittin	Reduce the risk of severe hemolysis and pain	Rheumatoid arthritis	Suppress the levels of pro-inflammation cytokines including IL-17 and TNF-α, and increase the percentage of regulatory CD4 T cells	Du et al., 2021a
Protein collagenase	Extend plasma half-life enhance stability	Transfer enzyme	Release the enzyme relatively fast to avoid its long exposure to water	Di Natale et al., 2021
Immunoglobulin G (IgG)	Enhance stability	Transfer IgG	Bioavailability has improved	Monkare et al., 2015
Lysozyme	Maintain enzyme activity	Anti-inflammatory anti-microbial	The protein integrity remained intact for three months in the three different polymeric MNs	Panda et al., 2021

**Table 8.** Summary of HA MNP delivery of hormone drugs.

Drug	Advantage	Adaptation disease	Results	Reference
Insulin	Avoid blood sugar sudden drops	Diabetes	Achieve a sustained but relatively fast release	Liu et al., 2012
Heparin	Avoid limitations of traditional injection, spontaneous hemorrhages	Thrombosis	Sense the thrombin level in blood vessels and autoregulate blood coagulation in a long-term manner	Zhang et al., 2017
Human growth hormone (hGH)	Avoid limitations of traditional injection Enhance bioavailability	hGH hyposecretion	Enhanced penetration of HA hGH conjugate through the dorsal skin of mice	Yang et al., 2012

**Table 9.** Summary of HA MNP delivery of vaccine drugs.

Therapeutic agent	Advantage	Adaptation disease	Results	Reference
Enterovirus 71 (EV71)	Avoid limitations of traditional injection	Hand-foot-and-mouth disease (HFMD)	Induced high level of antibody responses and conferring full protection	Zhu et al., 2016
Ovalbumin (OVA)	Avoid limitations of traditional injection	—	Elevated both humoral and mucosal antibodies with peak levels at four weeks	Kim et al., 2016
Cytotoxic T-cell epitope peptide	Avoid limitations of traditional injection	Melanoma	Inhibition of tumor growth by enhancing antigen-specific cytotoxic T-cell responses	Kim et al., 2019

**Table 10.** Summary of HA MNP delivery of genetic material drugs.

Therapeutic agent	Advantage	Adaptation disease	Results	Reference
siRNA	Enhance bioavailability	Skin disorders	Reduce expression of a targeted endogenous gene in a human skin xenograft model	Wang et al., 2020
siRNA	Enhance bioavailability	Human skin xenografts	Reduce expression of a targeted endogenous gene in a human skin xenograft model	Lara et al., 2012

## 6. Drug distribution of HA MNP

**Figure 8.**
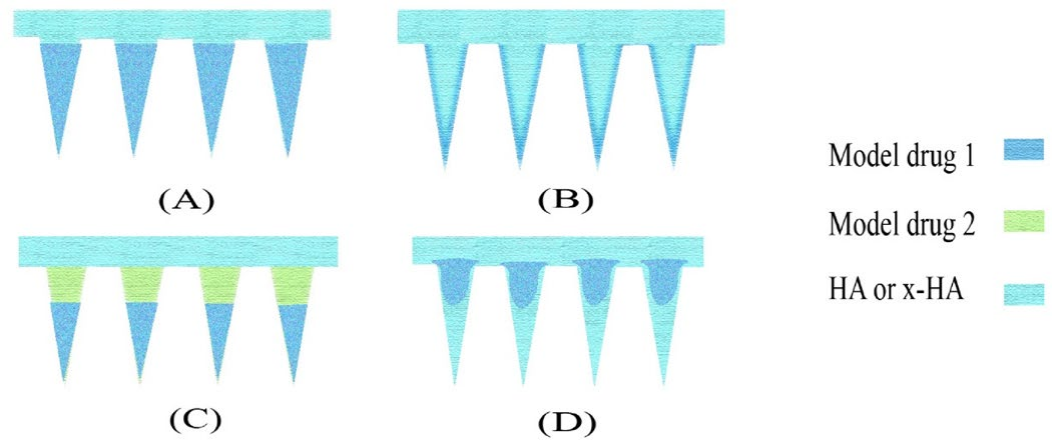
Four common drug distributions for HA MNP. (A) tip-loaded HA MNP (B) coating loading HA MNP. (C) on-demand layered HA MNP. (D) powder-laden HA MNP.

The drug distribution of HA MNP is similar to that of MNP prepared from other materials, but slightly different, due to the properties of HA such as rapid release and water absorption. The four most common types of drug distribution are tip-loaded HA MNP, Coating loading HA MNP (frozen spray-coating and dip-coating), on-demand layered HA MNP (tip and basal loading), and powder-laden HA MNP (Figure 8). Each type has its advantages and disadvantages. This section will be presented in conjunction with a variety of examples of HA MNP.

### 6.1. Tip-Loaded HA MNP

HA DMNP generally encapsulates the drug in the tip, which relies on the rapid dissolution of HA.

Tip loading can not only achieve rapid *in situ* release of loaded drugs and significantly improve instantaneous drug concentration, but also increase the bioavailability of the drug and avoid the incomplete release of rebound drug dissolution when penetrating the skin. As proof of concept, a tip-loaded HA DMNP made by Zhuang et al. can increase the drug delivery efficiency by 30% (Zhuang et al., 2020). Also, Korkmaz et al. developed tip-loaded HA DMNP that effectively delivered polymer-conjugated antibody inhibitors of TNF-α to human skin (Korkmaz et al., 2016). Such HA MNP for transdermal delivery is not only for humans only, but is equally applicable to animals. Recently, for example, a novel tip-dissolvable design called insertion response MNP (IMRMs) was proposed to reduce the time of insertion of HA MNP and to reduce discomfort during inoculation to animals (Choi et al., 2018a). Consisting of a dissolvable HA tip and a biocompatible polycaprolactone (PCL) base, the influenza virus vaccine antigen is successfully encapsulated on the HA tip. the PCL base is designed with a protruding wall on one side to enhance the mechanical resistance to non-axial stresses occurring during MNP insertion. During MNP insertion and retraction, its tip is instantaneously separated from the base. IMRMs utilize inter-material bonding forces to achieve tip separation rather than mechanical interlocking.

### 6.2. Coating loading HA MNP

HA MNP is water-absorbent, which is very conducive to freeze spraying and dip coating of drug solutions. Loading drug molecules onto the surface of HA MNP by freeze spraying or dip-coating reduces manufacturing time and cost improves the bioavailability of the drug and does not affect the mechanical properties of the MNP. However, the disadvantage is that the bottom drug may not reach specific sites. Insulin-laden bilayer expandable MeHA MNs were prepared by Ning et al. The number of layers could be increased by increasing the coating amount, and the coating process was experimentally shown not to affect the mechanical properties of HA MNs (Ning et al., 2020). Insulin could be effectively delivered to the blood circulation of mice to control blood glucose levels, and comparable efficacy to subcutaneous insulin injection was achieved, with glucose levels decreasing from ≈11.5 × 10^−3^ to ≈2.0 × 10^−3^ M within 60 min. Although the decrease in blood glucose concentration was slower than that of intradermal injection, hypoglycemia was prevented. Katsumi et al. used the dip-coating method to load alendronate (ALN) onto the tip portion of the HA needle for the treatment of osteoporosis. This design increased bioavailability to 96% without causing skin irritation. In a rat osteoporosis model, efficient delivery of ALN effectively inhibited the reduction of the growth plate. Briefly, the tip of MNs was immersed in 500 μL of a 5% HA solution containing 15 μg ALN for 2 seconds. The solubility tests showed the whole needle containing the drug has a high bioavailability of ALN (more than 90%), but the increase in plasma concentration tended to be delayed compared to subcutaneous injection. In contrast, needle-tip dipping (only the tip surface contains the drug) of ALN resulted in rapid drug release to inhibit osteoclast function and was effective in the prevention and treatment of osteoporosis (Katsumi et al., 2017).

### 6.3. On-demand layered HA MNP

On-demand layered drug delivery is a drug distribution based on delivery requirements, where drugs of different kinds are loaded at the tip and between the layers respectively for simultaneous delivery to different therapeutic sites for therapeutic purposes. Considering the different dissolution rates and drug release rates of HA and x-HA, it is a good choice for HA and X-HA as different matrix layers. Stratified drug delivery on demand has two advantages. The first advantage is layer-specific delivery and simultaneous delivery of different drugs to different sites, enabling one dose instead of multiple doses for complex comorbidities such as psoriatic arthritis. The second is that different laminar release rates can enhance the therapeutic effect, such as the release of bipolar antigen. Considering the different kinds of the loaded drugs and the need to achieve a certain mechanical strength, separate formulations of different layers of matrix material solutions should be designed to deliver different drugs to different sites of action simultaneously. For example, Yu et al. developed an HA MNP with an intermediate layer loaded with the immunosuppressant tacrolimus (TAC) and a tip layer loaded with the anti-inflammatory drug diclofenac (DIC), aiming to specifically deliver TAC and DIC to the skin and joint cavity while reducing psoriatic skin and arthritic joint lesions (Yu et al., 2021a). The matrix contains different layers (Table 11). *In vitro* and *in vivo* transdermal experiments showed that the intermediate layer released TAC at 100 μm of skin, while the tip layer delivered DIC into the joint cavity at 300 μm of skin. In animal model experiments, layered MNP significantly reduced swelling and inhibited TNF-a and IL-17A even better than DIC injection in other experimental groups (Chiu et al., 2018). Chiu developed a composite MNP consisting of a HA tip and a chitosan base for biphasic antigen release. After MNP implantation into the skin, the HA tip dissolves within the skin, rapidly releasing the encapsulated antigen and stimulating the immune system, while the degradable chitosan base remains in the dermis, prolonging antigen release for 4 weeks and further enhancing the stimulated immunity.

**Table 11.** Matrix material formula for each layer of on-demand layered HA developed by Yu et al.

Matrix solution	HA (wt/vol)	Dextran (wt/vol)	PVPK17 (wt/vol)	NIC (wt/vol)	Drug (wt/vol)
Tip-layer	10%	40%	10%	—	1% DIC
Inter-layer	10%	35%	15%	20%	0.1% TAC
Pedestal	30%	30%	10%	—	—

### 6.4. Powder-laden HA MNP

In the studies available so far, HA-filled MNP is mostly used for vaccines. The live attenuated BCG vaccine, the only licensed vaccine against tuberculosis in the world to date, must be given intradermally to be effective, which can cause severe skin inflammation and sometimes permanent scarring. HA MNP is minimally invasive and facilitates wound healing without producing any skin irritation or scarring. For example, Chen et al. developed the HA MNP, in which a deep hole is formed at the base of each needle and the BCG powder is loaded directly into the hole. It is delivered to the epidermis in a painless, noninvasive, and self-applicable manner. Upon insertion into the skin, the individual MNP shafts are dissolved by the interstitial fluid of the epidermis and upper dermis, and the powder is exposed to the epidermal tissue. The powder is drawn into the tissue fluid and slowly diffuses into the epidermis (Chen et al., 2017). Therefore, this soluble powdered MNP can be combined with many powdered vaccines or drugs without causing skin irritation.

## 7. Applications of HA MNP as an excellent drug carrier

**Figure 9.**
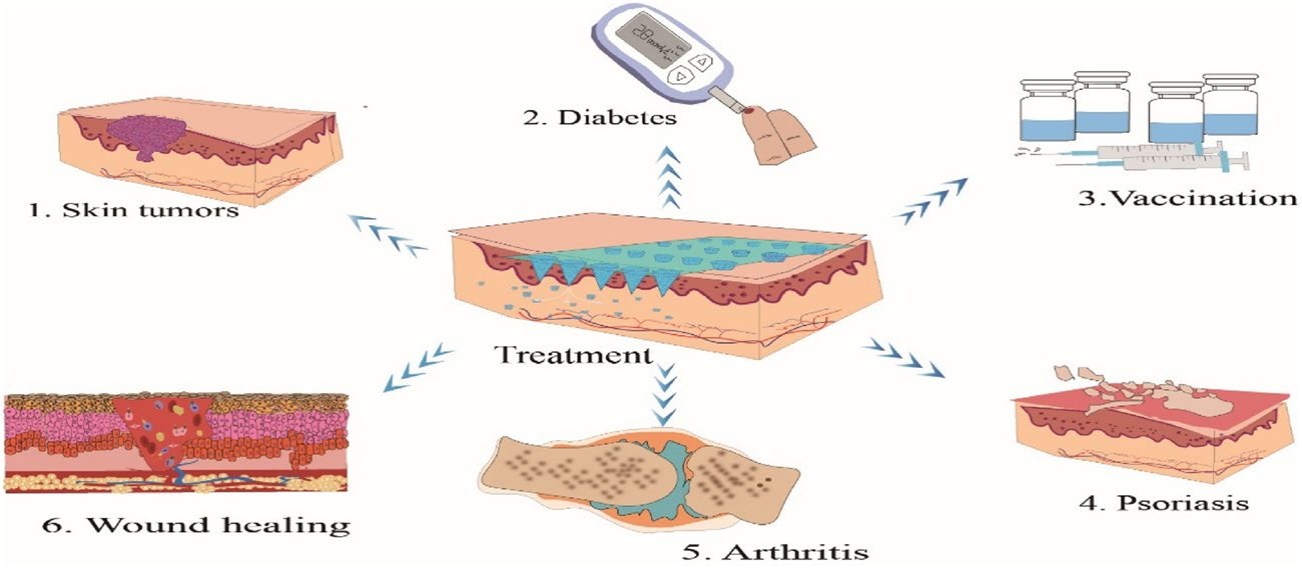
Application of HA MNP in treatment.

HA MNP has attracted plenty of interest in recent years from both the academics and pharmaceutical industries. Its biocompatible and biodegradable nature has led to its widespread use for both therapeutic and diagnostic drug designs against a number of diseases, and this section details both the therapeutic and diagnostic extraction aspects. The first aspect is its recent advances in skin tumors, diabetes, immunity, psoriasis, arthritis, wound healing, hyperuricemia, etc. (Figure 9). The second is some biomedical applications in extraction diagnostics and offline analysis of metabolites such as glucose and cholesterol.

### 7.1. Drug carrier in some specific diseases

#### 7.1.1. Targeting drug delivery HA MNP in skin tumors

Tumors have become a major threat to human health. Chemotherapy and photothermal therapy are the most widely used cancer treatment options in clinical practice but are associated with frequent and severe adverse effects that limit their clinical benefit. Among them, HA MNP has high safety levels in the treatment of superficial epidermal tumors compared to oral and injectable drugs (except in some cases of rash) (Wang et al., 2016). Currently, targeted treatment of superficial, metastatic, and SC tumors by delivery of 5-ALA, DOX, anti-programmed cell death protein 1 (aPD1), and death inducers is the most talked about technique. The drugs can easily cross the SC barrier and accumulate in the local peritumor skin network to achieve treatment of tumors. In addition, HA MNP has a significant advantage over MNP made of other materials in that it utilizes HA to achieve tumor-targeting activity by specifically interacting with the CD44 receptor, which is overexpressed on a variety of tumor cells. Last but not the least, the anaerobic and acidic environment provided by HA is also conducive to maintaining the long-term stability of 5-ALA and facilitating the transport of MNP (39). In another study in 2019, Zhu and colleagues loaded MNP with 5-ALA for photodynamic therapy of superficial tumors (Zhu et al., 2019). They were able to deliver about 368 µg of 5-ALA per delivery to pierce the skin to reach the targeted subcutaneous site, achieving better tumor treatment than the solution. Zhao et al. also prepared a fast DMNP based on photodynamic therapy (PDT) tip-loaded 5-ALA. This ensured the rapid release of the therapeutic dose at the site of action and improved the tumor inhibition efficacy to about 97% which was 1.5-fold compared to the injection group MNP group (Zhao et al., 2018). In addition, HYAL, which is overexpressed in the tumor microenvironment, can help HA enzymatic cleavage at the tumor site and activate drug release. In recent years, the combination of functional modification strategies, cooperative strategies, stimulus-response design strategies, built-in active dosing strategy (Lopez-Ramirez et al., 2020), and HA MNP has shown great promise in addressing these issues and overcoming the shortcomings of monotherapy and conventional therapies.

Functional modification strategy: Ye et al. used a strategy to design delivery checkpoint inhibitors aPD1 and 1-methyl-DL -tryptophan (1-MT) therapy to treat melanoma (Ye et al., 2016). Let HA covalently bind 1-MT to form an amphiphilic structure and self-assemble to build nanoparticles (NPs) encapsulating aPD1. This synergistic treatment facilitates specific targeting of the drug to accumulate to the tumor site. It was shown to overcome immunomodulatory mechanisms and enhance antitumor efficacy in the B16-F10 mouse melanoma model.

Collaborative strategy: Hao et al. developed HA MNP loaded with near-infrared light-responsive

5-fluorouracil (5-Fu) and indocyanine green (ICG) monomethoxy-poly (ethylene glycol)polycaprolactone (MPEG-PCL) nanoparticle (5-Fu-ICG-MPEG-PCL) for the inhibition of human superficial skin cancer (Hao et al., 2020). It was shown that the experimental group of MNP loaded with nanoparticles with combined infrared light response had the highest level of apoptosis and could not only thermally ablate tumors but also control the release behavior of 5-Fu in nanoparticles to achieve single-dose treatment of skin cancer. Moreover, Zhao et al. synergistically combined photothermal therapy (PTT) thermotherapy and chemotherapy with HAMNP to achieve low-dose and efficient drug delivery for tumor treatment (Zhao et al., 2021). A multimodal drug delivery system was prepared by incorporating a photothermal agent (CuS) into an imidazolate zeolite skeleton-8, which was functionalized with HA to co-deliver multimodal drugs. *In vivo* experiments showed that PTT thermotherapy could directly induce thermal ablation of tumor cells, and the synergistic effect with chemotherapy further enhanced the cell killing ability, demonstrating the superiority of HA MNP drug delivery. Yang et al. significantly promoted DOX accumulation in lymph nodes by combining nanotechnology (transfersomes) with HA MNP technology (Yang et al., 2019). The absolute bioavailability of this cooperative strategy was up to 79.9%, which was three times higher than the epidermal diffusion of DOX-T. *In vivo* experiments demonstrated significant killing of metastatic tumor cells present in lymph nodes. Chen et al. prepared HA DMNP using HA electrostatically combined with in situ self-assembled nano-micelles. Delivery of immunogenic cell death inducers (IR780) and autophagy inhibitors (chloroquine, CQ) effectively penetrated deep tumor tissues to treat superficial epidermal tumors (Chen et al., 2021b). *In vivo* results showed that the combination therapy effectively eliminated primary and distant tumors and inhibited tumor growth by remodeling the tumor microenvironment for more than 40 days. Dong et al. developed gold nanocages loaded with HA DMNP for the treatment of superficial skin tumors. It was shown that gold nanocages not only increased the mechanical strength of MNP but also showed good antitumor effects and safety in animal models (Dong et al., 2018).

#### 7.1.2. Smart HA MNP in diabetes

MNP technology is a new device with high drug delivery efficiency, easy administration, painless, low risk of infection, and has strong potential for clinical application, which is expected to become an effective drug delivery route for an insulin treatment of diabetes (Li et al., 2022). HA MNP has become an excellent choice for diabetes treatment because it facilitates wound healing, including micropores from injections and chronic wounds from diabetes. More novel at present is the smart insulin patch based on a closed-loop insulin delivery system synthesized from different glucose-responsive groups (Mo et al., 2014). These glucose-responsive moieties include phenylboronic acids (PBAs), GOx, and glucose-binding proteins (e.g., cutin A). For example, Fu and coworkers constructed a closed-loop insulin smart delivery system for type 1 diabetes treatment. This closed-loop insulin smart delivery system using HA MNP loaded with smart glucose-responsive PBAs-based mesoporous silica nanoparticles (Fu et al., 2022).First, the MNP tip layer was prepared by dispersing GMSN@Insulin@ZnO-PBA-2 into HA solution (0.5 g/mL). Then pure HA solution (1.0 g/mL) was added as the backing layer. The prepared HA MNP consisted of 15 × 15 pyramidal needles with a height of approximately 500 µm. The PBAs formed reversible dynamic covalent bonds with cis-1,2-diols and cis-1,3-diols of sugar. During hyperglycemia, the linkage between PBA-2 and glucosamine on mesoporous silica nanoparticles is severed, to facilitate insulin release.

*In vivo* studies showed that mice treated with HA MNP had a longer glucose regulation time of 3.5 h. Intraperitoneal injection of glucose (1.5 g/kg) was able to quickly return to normal levels within 30 minutes and maintain similar levels in healthy mice, with good glucose control and avoiding hypoglycemia (Yu et al., 2015). Yu et al. used HS-HA self-assembled with NI to form glucose-responsive vesicles encapsulating insulin and GOx and then loaded the vesicles in HA MNs tips (Yu et al., 2015). This responsive MNP rapidly triggered vesicle dissociation in a hyperglycemic state, which in turn released insulin. Compared with the control group, the glucose level of vesicle-loaded MNP rapidly decreased to about 200 mg/dL within 1 hour and was maintained at a normal glucose concentration of about 200 mg/dL for the next 3 hours, effectively reducing the risk of hypoglycemia. Lee and coworkers designed a sweat glucose monitoring device in combination with feedback transdermal drug delivery MNP. The use of electrochemical analysis provides a new avenue for noninvasive glucose monitoring through multistage and precise control of drug release. The type 2 diabetes drug (metformin or chlorpropamide) used for feedback transdermal therapy is loaded on two different temperature-sensitive phase change nanoparticles. These nanoparticles were embedded in HA hydrogel MNP coated with a phase change material to reduce MNP dissolution. Statistical analysis confirmed that sweat glucose levels measured by wearable and disposable sweat glucose sensors were consistent with glucose levels measured by commercial glucose meters (Lee et al., 2017). Hypoglycemia is a serious and potentially fatal complication experienced by patients with insulin-dependent diabetes. High levels of growth-inhibitory hormone secreted by δ-cells further inhibit glucagon secretion, thereby counteracting excess insulin. Therefore, GhavamiNejad et al. developed a MeHA MNP to transdermally deliver growth inhibitor receptor type 2 antagonists to prevent hypoglycemia (GhavamiNejad et al., 2022). Molecular dynamics (MD) simulation methods showed that MeHA stabilized the structure of the peptide PRL-2903, and the antagonist-containing MNP successfully prevented insulin-induced hypoglycemia within 2.5 h after overdose. In conclusion, MNP technology offers a new potential therapy for the treatment of diabetes.

#### 7.1.3. HA MNP in vaccination

With the 2019 outbreak of widespread coronavirus disease (COVID-19) infection, it has become particularly important to develop many safe and effective vaccination options to prevent infection with coronavirus and influenza. Traditional injectable vaccination has a number of problems, such as pain, the need for medical personnel or technology, needle-related illness or injury, and storage and transportation. Improving the immune response to vaccines and minimizing the need for repeat vaccinations remains a challenge for clinical vaccination. Transdermal immunization (TCI) is an attractive alternative route of vaccination. The skin is considered an ideal target for vaccine delivery, with MNP penetrating the SC and introducing antigens into specific areas of the skin to maximize interaction with resident antigen-presenting cells (APCs), and TCI-induced immune responses have been reported to be equivalent to or higher than those induced by intramuscular injections. In addition, due to the higher number of APCs in the skin, resulting in a stronger immune response at lower antigen levels, only a smaller dose of vaccine is required for dermal vaccination compared to conventional intramuscular (IM) vaccination. As a result, MNP as a vaccine carrier has become a very active field in recent years. Scientists demonstrate effectiveness of HA DMNP for vaccination prophylaxis (Tetanus toxoid (TT), diphtheria toxoid (DT), SE36, OVA). Back in 2012, Matsuo and colleagues reported the potential advantages of using TT, DT, and SE36 as antigen to detect the TCI system. And they compared the immune effect of the TCI system with conventional immune systems such as subcutaneous immunity (SCI), intradermal immunity (IDI), intramuscular immunity (IMI), and intranasal immunity (INI). Matsuo prepared two types of MNs arrays, 300 μm and 800 μm, and fixed them on a certain area of adhesive film to form MNP, demonstrating that this TCI system could replace the conventional injection system (Matsuo et al., 2012b). In the same year, he demonstrated that a HA DMNP could simply, safely, and effectively enhance the protective immune response of each of these vaccine antigens (Matsuo et al., 2012a). Later, Kim et al. demonstrated an effective, noninvasive transdermal vaccination by using HA MNP as a vaccine carrier combined a non-exfoliative laser adjuvant. The combination of HA significantly improved vaccination efficiency (Kim et al., 2016). Studies have shown that topical application of HA-OVA conjugate significantly increased humoral and mucosal antibodies, peaking at week 4 and elicited a strong immune recovery response at week 8, while pretreatment with a non-exfoliative laser adjuvant allowed for a strong immune response at a low dose of vaccine. Notably, Chiu et al. developed a composite MNP for biphasic antigen (Chiu et al., 2018). Studies have shown that most of the OVA loaded on the HA tip is released and dissolved in the skin within 7 days, while only 35% of the OVA is released from the chitosan matrix, which can retain the antigen at the delivery site and prolong the antigen exposure time up to 4 weeks. Further enhancement of the stimulated immunity will be facilitated. Antibody response induction in this manner is significantly higher than double conventional agent treatment or double dose subcutaneous vaccination.

#### 7.1.4. Targeting drug delivery HA MNP in psoriasis

Psoriasis is an immune-mediated chronic inflammatory skin disease with clinical manifestations such as erythema, scales, and inflammatory plaques on the skin. Traditional treatment regimens include the use of high doses of steroids, methotrexate (MTX), cyclosporine, and other drugs that have toxic effects such as suppression of the immune system and damage to vital organs. To overcome these drawbacks, MNP is being developed to replace conventional therapies. HA is one of the most widely used polymers in the manufacture of MNP for psoriasis because not only is it an endogenous substance in the skin’s surface and dermis with the ability to retain cellular moisture and improve the healing of psoriatic lesions, but also CD44 is highly expressed in psoriatic skin. Studies have found that HA can improve psoriasis treatment by inhibiting CD44 and PKCa interactions to achieve anti-allergic effects. For example, in a 2016 clinical study on HA MNP, St. Vincent’s Hospital evaluated the efficacy of intensive transdermal administration of HA MNP for the treatment of psoriatic plaques, enrolling 20 patients with psoriasis. The patients’ six psoriatic plaques were randomly divided into an HA MNP group, a patch group, and a control group. All lesions will be treated for 2 weeks. All lesions in the control group were be treated with calcitriol dipropionate betamethasone ointment only. All lesions in the patch group will be treated with a calcitriol dipropionate betamethasone ointment patch to exclude the effect of the patch. All lesions in the HA MNP group were treated with topical calcitriol betamethasone dipropionate ointment plus MNP. Experimental results showed that the therapeutic effect of HA MNP group was better than the other two groups.

Du et al. loaded MTX into HA DMNP, and *in vitro* studies showed that drug activity was not affected in the MNP structure (Du et al., 2019), and the proliferative capacity of HaCaT cells was significantly inhibited when MTX concentration was greater than 1 μg/mL. Compared with the oral double-dose group, the MNP group significantly reduced psoriatic dermatitis in mice. It could reduce systemic absorption and contribute to the reduction of adverse systemic reactions. Psoriatic arthritis is complex psoriatic comorbidity that requires a combination of different drug treatments, but Yu and colleagues provided an alternative to multiple drug delivery for the treatment of psoriatic arthritis comorbidity by delivering different drugs simultaneously to different sites of action of psoriasis (Yu et al., 2021a). Particularly clever use of layered DMNP specifically delivered the immunosuppressant TAC and the anti-inflammatory drug DIC to the skin and joint cavity. Compared with other groups, the layered DMNP demonstrated sustained therapeutic effects, effectively reducing the area and epidermal thickening of psoriasis, while reducing joint inflammation.

#### 7.1.5. HA MNP for collaborative therapy in rheumatoid arthritis

Rheumatoid arthritis (RA) is an autoimmune disease characterized by chronic synovial inflammation. It is accompanied by swelling, stiffness, and erosion of the joints. Severe rheumatoid arthritis can lead to functional impairment, organ failure, and infection. Oral or injectable non-steroidal anti-inflammatory drugs (NSAIDs) or glucocorticoids are usually used to reduce inflammation and slow the progression of RA by interfering with inflammation-related pathways. However, gastrointestinal irritation, and hepatic first-pass effects can lead to a significant decrease in drug bioavailability.

Du and coworkers prepared HA MNP loaded with melittin, which significantly reduced paw thickness in both the pure HA-loaded melittin and MeHA-loaded melittin groups compared to the subcutaneous injection group. Effectively, the symptoms of RA were reduced. However, the MeHA-loaded melittin group not only had melittin slow-release properties but also provided better protection against melittin than the other two groups. In addition, the application of HA MNP suppressed the levels of pro-inflammatory cytokines IL-17 and TNF-α (Du et al., 2021a). Cao et al. developed MeHA DMNP to deliver the biomacromolecule TNF-α inhibitor etanercept, and experiments demonstrated that the activity of etanercept was not affected in the HA MNP. Compared to the control group, the foot swelling rate in the MNP group decreased from 1.68 to 1.44 within 10 days, indicating an anti-inflammatory effect and a decrease in serum TNF-α and IL-6 concentrations. HA MNP showed excellent bioequivalence and higher compliance (Cao et al., 2019). This noninvasive and highly effective MNP lays the foundation for the treatment of autoimmune diseases such as RA.

#### 7.1.6. HA MNP for collaborative therapy in wound healing

To comprehensively assess and care for chronic wounds, the TIME (Tissue, Infection, Moisture, and Wound Edge) system was described as the standard of care in 2002. Emphasis is placed on the following concerns in promoting wound healing: removing necrotic tissue, eliminating bacteria, maintaining wound moistures while aspirating excess exudate, and promoting healthy cell growth from the wound edges. The ideal wound dressing material should have anti-inflammatory activity, antibacterial, and strong regenerative capabilities. Wounds are divided into chronic wounds (diabetic ulcers, etc.) and acute wounds. Researchers have recently demonstrated that the complex environment of chronic wounds may reduce the local availability of applied treatments. MNP can be used to improve healing by increasing transport efficiency. HA, a highly hydrophilic polymer and itself a component of ECM, significantly promotes angiogenesis, and collagen deposition, reduces wound inflammation, stimulates differentiated macrophages, and plays an important role in wound healing. x-HA MNP can be used for chronic wound healing. Pure HA MNP can be used for acute wound healing.

Recently Ma et al. proposed a multifunctional novel core-shell HA MNP to promote wound healing in diabetic chronic wounds with antioxidant, anti-inflammatory, and pro-angiogenic properties (Ma et al., 2022). Fe-MSC-derived artificial nanocapsules (Fe-MSC-NVs) were encapsulated in the inner HA core of MN tips to accelerate angiogenesis. Iron nanoparticles significantly enhanced the expression of therapeutic cytokines by MSCs-NVs. Polydopamine (PDA) nanoparticles were encapsulated in MNs tip shells made of MeHA to inhibit ROS-induced inflammatory responses and increase mechanical strength. PDA proved to be an effective antioxidant due to its large number of reducing functional groups such as catechol and imine. It was shown to significantly promote healing in a 1.5 cm diameter full skin wound model. In addition, the HA MNP is biodegradable and biocompatible, making drug delivery and absorption more efficient, convenient, and safe, and the drug activity is not affected in the MNP matrix. Recently, Yao et al. prepared a MeHA hydrogel MNP encapsulated with Zn-MOF that possesses ideal wound dressing properties (Yao et al., 2021). It has the advantages of being antibacterial, trauma friendly, high ductility, degradability, and avoidance of secondary damage. Firstly, MeHA hydrogel MNP loaded with Zn-MOF can continuously and stably release zinc ions, and Zn^2+^ can disrupt the integrity of bacterial membranes, and oxidative stimulation eventually leads to bacterial death. Secondly, the LWV-HA generated by MeHA hydrolysis significantly accelerates the proliferation/migration and angiogenesis of keratin-forming cells by inducing the production of VEGF and adhesion molecules. In addition, MeHA hydrogel MNP provides a larger specific surface area to interact with wounds, and experimental results showed that the MeHA hydrogel MNP group loaded with Zn-MOF significantly inhibited the amount of IL-6 secretion, and the circular wounds on the back of rats almost healed after 9 days. Arshad et al. used HA and PVP as MNP matrices loaded with macrolide azithromycin to treat wounds infected, and by day 5 the wounds were completely healed, the S. aureus biofilm disappeared, and skin structures (hair follicles, dermis) regenerated. The results confirmed the antibiofilm effects of the prepared HA MNP (Arshad et al., 2021). Park and coworkers prepared an antimicrobial HA MNP consisting of green tea (GT) extract. It was used for efficient delivery of GT, which reduced bacterial growth and improved the healing process of skin wounds. *In vitro* release studies showed that the MNP could release GT continuously (Park et al., 2014), the degradation rate and release kinetics could be regulated by controlling the concentration of HA.

#### 7.1.7. HA MNP in hyperuricemia

Treatment of hyperuricemia with conventional drugs has limited clinical efficacy and can cause serious side effects such as gastrointestinal irritation, liver and kidney failure, and bone marrow suppression. Recently, some researchers use the HA DMNs delivery systems for percutaneous administration of hyperuricemia. Hao and colleagues integrated Uricase&HRP-CaHPO4 nanoflowers with HA DMNs to produce Uricase &HRP-CaHPO4@HA MN (Hao et al., 2019). Uricase&HRP-CaHPO4@HA MN has excellent skin penetration and is as effective as intravenous administration in reducing uric acid levels in the body. However, intravenous uricase may produce a large amount of hydrogen peroxide and cause serious side effects. Chen et al. first proposed that DMNs loaded with allopurinol (AP) were administered percutaneously to slowly release AP to improve its bioavailability (Chen et al., 2021a). DMNs with sufficient mechanical strength were prepared by suspension casting with precise dose control. Pharmacodynamic experiments confirmed the sustained release and anti-hyperuricemia effects of DMNs coated with AP. In addition, studies have shown that DMNP are well tolerated. In conclusion, the HA DMNP have a small first-pass effect on the liver and high patient compliance, which may provide a new option for constructing a novel MNP delivery system and exploring the clinical treatment of hyperuricemia.

### 7.2. HA MNP for extraction and analysis of biomarkers

#### 7.2.1. Extraction of ISF

Point-of-caretesting (POCT) diagnostic technology plays an important role in disease diagnosis and prognosis, rapid determination of treatment, physiological status monitoring, personalized medicine, etc. Traditional methods such as suction blister, microdialysis, and open-flow micro-perfusion generate pores or pathways in the skin. ISF is obtained by capillary action or vacuum. Problems include time-consuming, troublesome, patient-unfriendly, requiring medical expertise, and specialized equipment. ISF accounts for about 45% of the volume fraction of human skin. ISF is a promising source of body fluids that can be used to understand tissue physiology by monitoring biomarkers and cellular components, which in turn report on the physiological state of patients. ISF is formed by capillary filtration of blood, it has similar components to plasma and changes with physiological changes. Now, an emerging alternative to the traditional method is hydrogel HA MNP. The natural water absorption of HA makes it an ideal candidate for rapid ISF extraction. Extraction of ISF with HA MNP is becoming an emerging method for the diagnosis and prognosis of diseases (Li et al., 2022).

#### 7.2.2. Application of MeHA hydrogel MNP

MeHA hydrogel MNP is an expansive MNP that can rapidly extract ISF. MNP is made by using MeHA as an MNP matrix, adding an appropriate photo-initiator, and further crosslinking through ultraviolet irradiation. Due to its strong hydrophilicity, covalent crosslinking network, and mechanical strength, MeHA MNP can maintain structural integrity in the expansive hydration state after use and will not remain in the skin. With no need for additional assistive devices, ISF can be extracted quickly and in one step in a short time after the thumb, pressure penetrates the skin. After extraction, it can be analyzed and detected by simple processing. Patients can self-test to avoid cross-infection in the hospital and reduce occupational exposure of health care workers.

**Figure 10.**
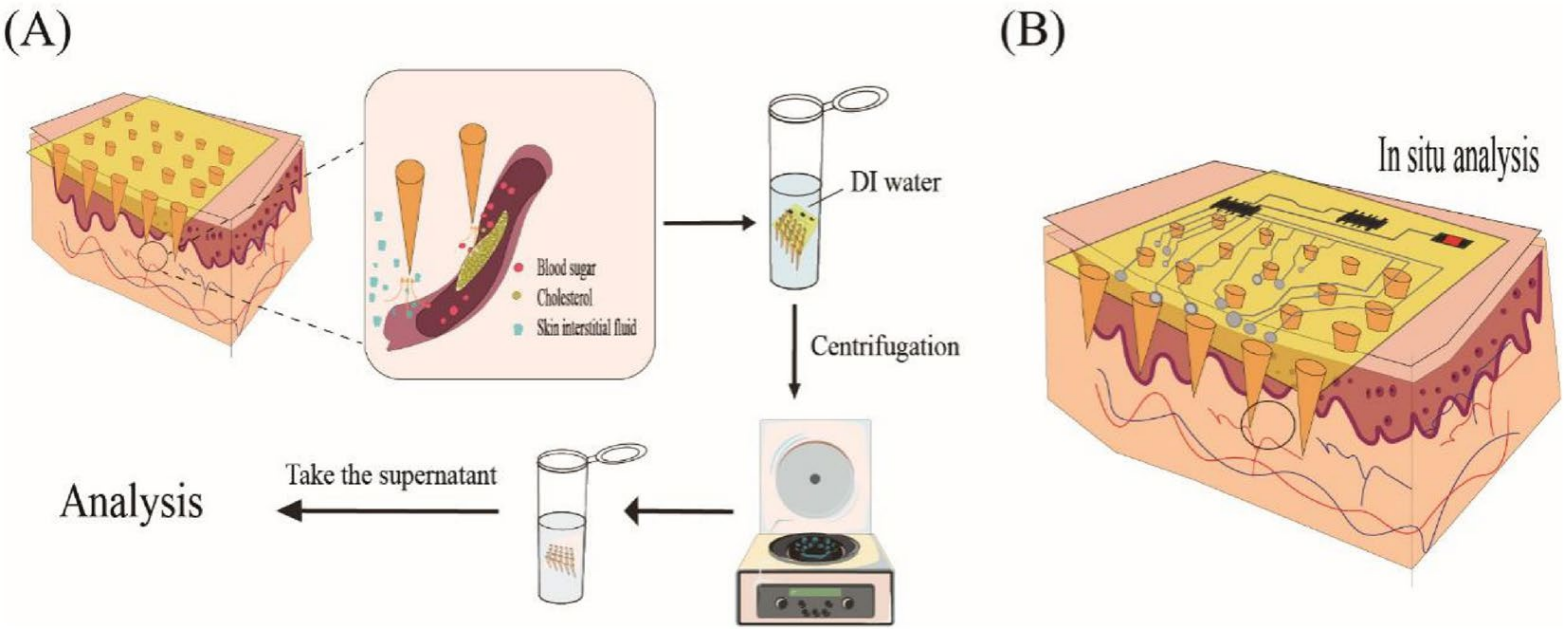
Two patterns of HA MNP extraction diagnosis. (A) ISF was extracted from the MeHA hydrogel MNP and then analyzed. (B) Detection chip and the MeHA hydrogel MNP integrated device for in situ analysis.

In recent years, HA MNP has attracted more and more interest in extraction and diagnosis and has been widely used in many cutting-edge biomedical fields. Chang et al. reported a MeHA hydrogel MNP that extracted about 1.4 mg of ISF within 1 min and was structurally intact without skin residue (Figure 10A) (Chang et al., 2017). The extracted ISF metabolites can be efficiently recovered from the patch by high-speed centrifugation, and the levels of blood glucose and cholesterol detected by MNP have the same values and trends as those measured by conventional glucose meters and cholesterol measurements. Although the extraction time of this method is short, the subsequent recovery analysis time is long, which may affect the detection results. To overcome this challenge, Zheng et al. prepared a MeHA hydrogel MNP with an osmotic agent (maltose) and very cleverly placed an electronic glucose sensor directly on the backing layer of the MNP to analyze in situ MNP containing glucose (Figure 10B) (Zheng et al., 2020). ISF was not centrifugally recovered from MN to the aqueous solution. In addition, a hydrogel MNP with osmotic power can extract ISF faster. During extraction, the penetrant dissolves in the matrix and provides osmotic pressure, increasing the diffusion of ISF from the skin to the hydrogel matrix. MeHA MNP directly measures pig skin glucose concentration *in vitro* through integration with an electronic glucose sensor, without any post-processing methods. MeHA hydrogel MNP makes it possible to simultaneously extract ISF and release pre-loaded drugs during swelling. Puigmal et al. cleverly used MeHA hydrogel MNP to simultaneously deliver drugs and extract tissue cells, locally delivering the chemokine CCL22 (to enhance regulatory cell (Treg) recruitment) and the cytokine immunomodulator IL-2 (to maintain *in vivo* Treg homeostasis), which can enhance the immune tolerance environment during allogeneic skin grafting (Puigmal et al., 2021). After ISF sampling, the presence of Tregs in allograft skin biopsy tissues was detected by adding reducing agents to break the 3 D structure of MNP and then by flow cytometry. A higher proportion of Treg was found in CCL22+ IL-2 (10 ng) MNP treated allograft skin grafts compared to blank MNP controls, consistent with the distribution of Treg observed in ISF recovered after MNP sampling. It was confirmed that the prepared MNP reflected the immune status of the allograft skin. It was shown that this strategy not only helps to locally modulate the immune system but also helps to monitor the rejection of skin grafts to treatment after transplantation.

HA MNP-based diagnosis can be divided into three modes according to the location of diagnosis results: (1) ‘Off device’, HA MNP device is only used for transdermal sampling of biological fluid, and the sample is transferred to a central laboratory for testing; (2) ‘On device’ integrates the miniaturized analyzer and MN device without sample transfer; (3) ‘On MNs’, each MN installed in the HA MNs device can be used for *in vivo* biomarker collector or analyzer. Both ‘On device’ and ‘ON MNs’ modes can be used to perform rapid diagnostic tests at the medical point. Table 12 lists some examples of HA MNP for diagnostic applications.

**Table 12.** Multiple examples of HA MNP used to extract diagnostics.

Analyte	Sensor structure	Detection method	Application	Test subject	Detection site	Reference
miRNA and Cu^2+^	GelMA-MeHA MNP	Catalytic hairpin assembly (CHA) and carbon quantum dots (CQDs)	Breast cancer	Fresh porcine cadaver skin model	On MNs	Li et al., 2022
CD8^+^, CD4^+^, and FOXP3^+^	HA hydrogel crosslinked with 8-arm-PEG-NHS MNP	Polymerase chain reaction (PCR)	Monitoring of Immunotherapy Efficacy	Mouse	Off device	Puigmal et al., 2021
Glucose	MeHA MNP	Electronic Glucose Sensor	Diabetes	Mouse	On device	Zheng et al., 2020
Glucose and cholesterol	MeHA MNP	Glucose assay kit and cholesterol quantitation kit	Diabetes or Cardiovascular disorders	Mouse	Off device	Chang et al., 2017
Glucose, lactate, cholesterol, and pH	MeHA MNP	Wax-patterned and sensing-reagent-decorated test paper	Metabolic diseases	Mouse	On MNs	Zhu et al., 2022

MNP-based POCT devices have attracted increasing attention from researchers due to their great potential to detect various analytes in a minimally invasive manner (Liu et al., 2020a). However, in terms of extraction and diagnosis, HA MNP still faces three challenges. First, HA MNP can only be used for the extraction of superficial body fluids, but cannot be used to extract substances from the internal body for diagnosis, including both solid substances and fluids. Changing the shape and usage of MNs may be beneficial to solve the problem. However, traditional diagnostic methods are still the main means to extract internal markers of the body, such as amniotic fluid and lumbar puncture, and tumor biopsy. Second, although the HA MNP achieves nearly painless and noninvasive extraction and can improve patient compliance, merely in blood glucose tests nowadays, only a few composite patches that integrate extraction and diagnosis were reported (Lee et al., 2017; Liu et al., 2020c). The further diagnosis of most biomarkers requires the help of specialized equipment, and patients cannot obtain the diagnosis results by themselves. Therefore, it is the future direction of MNP application in extraction and diagnosis to develop intelligent patches integrated with extraction and diagnosis combined with high and new technology. One of the biggest challenges in this field is to get tiny devices that can bind to HA MNP and test for a particular biomarker. Lee et al. used soft bioelectronic technology on human skin to perform electrochemical analysis of glucose concentration in sweat, transdermal delivery of appropriate amounts of metformin (or chlorpropamide), and noninvasive blood sampling to monitor blood glucose (Lee et al., 2017). This design enables precise control of blood glucose levels in patients responding to drug release in a multistage, multispatial mode. Drug delivery can be thermally controlled in a multistage manner. The MNP assembled on the multichannel thermal actuator can be periodically replaced with new ones. These patch-type devices are fabricated on a processed substrate and then transferred to print onto a thin silicone patch. The sensor is fabricated on a thin polyimide (PI) substrate to achieve graphical electrochemical functionalization. Above the critical humidity, glucose, pH, and temperature, sensors begin measuring to determine the relevant blood glucose level. PH and temperature sensors can correct potential errors by enzyme-based glucose level measurements in real-time. During hyperglycemia, feedbacks from heat-driven control in the MNP activate transdermal delivery of metformin. Such a composite patch integrated with individualized medicine may be the future research direction of MNP for extraction and diagnosis. To achieve this goal, it is essential to integrate MNP with electronic microchip components. To fully apply microchip MNP to the clinic, it is necessary to improve the existing MNP manufacturing technology and achieve the large-scale production of MNP. In addition, the accuracy and sensitivity of the electronic sensors and effector elements inside the microchip will need to be tuned through improved programming and wireless communication technologies.

## 8. Conclusions and prospects

MNs systems, as cutting-edge technology for TDDS, can largely overcome the limitations of oral drug delivery, subcutaneous injection, and other transdermal drug delivery methods. HA as a recognized biocompatibility natural polymer has been widely designed and prepared for MNP, and HA MNP has strongly promoted the development of a transdermal drug delivery system for local or systemic drug delivery because of its easy preparation, biodegradability, biocompatibility, and easy to modify. As a result, the data are given on the official website of Chinese patents also accounts for more than one-tenth, with 566 applications. In the past decade, research on HA MNP has made significant progress in transdermal drug delivery, disease diagnosis, and monitoring.

In the future, we envision that HA MNP will be used for cell delivery, such as stem cells for organ repair and regeneration, pancreatic islet cells, immune cells, etc. In 2021, Xu et al. from the City University of Hong Kong in China combined frozen HA MNP with cell therapy. Designed to load and transport live cells into the skin, the therapy can facilitate a series of minimally invasive cell delivery cell therapies (Chang et al., 2021). In addition, since a composite MNP loaded with biphasic antigen has been developed by Chiu et al., there is reason to believe that in the future HA MNP could replace small doses of vaccine injections. As a proof of concept, the HA material can stabilize some of the antigens, and degradation products are safe and promote needle wound healing. Most importantly, since HA activates immune pathways, we hypothesize that HA MNP delivery of vaccines would have a synergistic effect. Also, we envision future applications for the extraction of blood biomarkers such as antibodies to hepatitis B antigens, HIV antibodies, and hepatitis C antibodies.

It is worth mentioning that the preparation of HA MNP is very time-consuming. This is the main reason to limit the large-scale use of HA MNP. Preparation methods such as micro-molding, laser cutting, photolithography (Lee et al., 2010), and wet and dry etching (Roh et al., 2022) have now been developed. But industrial production requires a more simple method. Recently, 3 D printing has been introduced as a powerful MNP fabrication strategy. Ouyang et al. (Ouyang et al., 2020) investigated bio-inks represented by MeHA, which can meet the physicochemical requirements of printing and provide an ideal environment for encapsulating cells. Petta D et al. summarized the recent research progress of HA-containing bio-inks for 3 D printing (Petta et al., 2020), giving us a lot of inspiration for choosing the right bio-ink to prepare HA MNP. We hope that in the future, we can find the perfect bio-inks for HA MNP printing.

This review proposes some limitations and feasible recommendations. Although HA MNP has attracted attention from scientists, its biomedical applications are still in their infancy. Only a certain amount of experimental studies have been conducted in the medical, pharmaceutical, and cosmetics industries. More clinical trials will be needed soon to provide solid evidence, and we hope the scientific community will come up with an agreed guideline. It is hoped that this initiative will standardize all phases of formulation development, optimize *in vivo* assessment models, and accelerate the translation from clinical trials to the clinic. In addition, its high cost and the challenge of registering it as a commercial drug is a major difficulty in bringing it to market. We hope this review will appeal to many researchers, inform the selection of MNP materials in future studies, and facilitate the development of HA MNP technologies for biomedical applications. Eventually, HA MNP will move toward large-scale production and practical use to bring convenience to treating various diseases.
